# Advances in Hodgkin Lymphoma Treatment: From Molecular Biology to Clinical Practice

**DOI:** 10.3390/cancers16101830

**Published:** 2024-05-10

**Authors:** Corrado Benevolo Savelli, Matteo Bisio, Luca Legato, Filippo Fasano, Elisa Santambrogio, Maura Nicolosi, Deborah Morra, Carola Boccomini, Roberto Freilone, Barbara Botto, Mattia Novo

**Affiliations:** Hematology Division, A.O.U. Città della Salute e della Scienza di Torino, C.so Bramante 88, 10126 Turin, Italy; mbisio@cittadellasalute.to.it (M.B.); llegato@cittadellasalute.to.it (L.L.); ffasano@cittadellasalute.to.it (F.F.); esantambrogio@cittadellasalute.to.it (E.S.); manicolosi@cittadellasalute.to.it (M.N.); demorra@cittadellasalute.to.it (D.M.); cboccomini@cittadellasalute.to.it (C.B.); rofreilone@cittadellasalute.to.it (R.F.); bbotto@cittadellasalute.to.it (B.B.)

**Keywords:** Hodgkin Lymphoma, immunochemotherapy, immune checkpoint inhibitors, anti-CD30 drugs, T-cell regulation, CAR T-cells, epigenetic modulation

## Abstract

**Simple Summary:**

Classical Hodgkin Lymphoma is a blood cancer, accounting for 0.5% of all new cancer diagnoses. Despite high cure chances, approximately 20% of patients are refractory to frontline treatment or relapse thereafter. Treatment strategies for relapsed/refractory patients have progressively lower chances of inducing a persistent complete remission. Therefore, great efforts are being made to further improve rates of response of frontline therapy, as well as to explore the efficacy of new compounds and different drug combinations. The present review aims to summarize these efforts, offering an overview of recent advances and future perspectives in the field.

**Abstract:**

Classical Hodgkin Lymphoma (cHL) is a highly curable disease, but around 20% of patients experience progression or relapse after standard frontline chemotherapy regimens. Salvage regimens followed by autologous stem cell transplants represent the historical treatment approach for these cases. In the last decade, with the increasing understanding of cHL biology and tumor microenvironment role in disease course, novel molecules have been introduced in clinical practice, improving outcomes in the relapsed/refractory setting. The anti-CD30 antibody-drug conjugated brentuximab vedotin and PD-1/PD-L1 checkpoint inhibitors represent nowadays curative options for chemorefractory patients, and randomized trials recently demonstrated their efficacy in frontline immune-chemo-combined modalities. Several drugs able to modulate the patients’ T-lymphocytes and NK cell activity are under development, as well as many anti-CD30 chimeric antigen receptor T-cell products. Multiple tumor aberrant epigenetic mechanisms are being investigated as targets for antineoplastic compounds such as histone deacetylase inhibitors and hypomethylating agents. Moreover, JAK2 inhibition combined with anti-PD1 blockade revealed a potential complementary therapeutic pathway in cHL. In this review, we will summarize recent findings on cHL biology and novel treatment options clinically available, as well as promising future perspectives in the field.

## 1. Introduction

Classical Hodgkin Lymphoma (cHL) is a rare disease accounting for 10% of all lymphomas and 0.5% of all new cancer diagnoses, and it is characterized by a bimodal distribution, affecting mostly young adults and people aged over 50 [1]. Despite cHL being considered a highly curable disease, approximately 20% of patients fail the standard frontline chemotherapy, mostly represented by the schemes doxorubicin, bleomycin, vinblastine, and dacarbazine (ABVD) or escalated bleomycin, etoposide, doxorubicin, cyclophosphamide, vincristine, procarbazine, and prednisone (eBEACOPP) [2,3]. Salvage strategies consisting of high-dose chemotherapy followed by autologous stem cell transplant (ASCT) represent the standard of care for transplant-eligible patients and are curative in around 50% of cases [4]. In the recent era, building on expanded knowledge of cHL biological complexity, novel drugs have been developed in order to improve outcomes for chemorefractory patients.

Hodgkin lymphoma is characterized by peculiar pathological features: the typical Hodgkin and Reed Sternberg (HRS) tumor cells represent less than 5% of the involved tumor tissue and are surrounded by a complex tumor microenvironment (TME) consisting of a varied polyclonal immune background that includes T helper cells, natural killer (NK) cells, dendritic cells, tumor-associated macrophages (TAMs), mast cells, eosinophils, stromal cells, plasma cells, fibroblasts and other elements [5,6]. HRS malignant cells display a characteristic immunophenotype with typical CD30 positivity and release several cytokines, including a number of interleukins, transforming growth factor ß (TGF-ß) and granulocyte-macrophage colony-stimulating factor (GM-CSF), which all contribute to TME modulation and immune control evasion [7,8].

CD30 protein expression and the programmed cell death protein-1 receptor (PD-1) and ligands (PD-L1/PD-L2) immune modulatory pathway have proven to be relevant for tumor development and survival and represent key targets for novel treatment strategies [9,10]. Given the importance of the immune system in tumor control, a number of strategies aimed at immune system modulation, reconstitution and enhancement are under development, such as chimeric antigen receptor (CAR) T-cells, NK cell-targeting bispecific antibodies and novel immune checkpoint inhibitors. Finally, the study of the aberrant epigenetic mechanisms involved in oncogenesis led to investigations on epigenetic modulating agents as a potential treatment strategy for cHL.

In this review, we provide an overview of novel, approved and emerging treatment strategies for cHL driven by increasing knowledge of biological features of the disease and its pathophysiologic molecular pathways. With this purpose, a literature research including papers, international oncology guidelines and abstracts derived from main international conference proceedings up to February 2024 has been conducted.

## 2. CD30 and CD30 Targeted Therapies

CD30 antigen or Ki-1 is a 120-kDA glycosylated type I transmembrane protein belonging to the Tumor Necrosis Factor Receptor Superfamily encoded by a gene located on chromosome 1p36 [11,12]. The interaction between CD30 and its ligand has pleiotropic biological effects depending on the involved cell type and other stimuli, ranging from providing proliferation and survival signals to promoting cell death [13,14]. CD30 is expressed on multiple lymphoid cells, including activated B and T/NK cells, virus-infected lymphocytes and malignant cHL and Non-Hodgkin Lymphoma (NHL) cells [11,12]. The low or absent expression of CD30 on healthy tissues besides activated B and T/NK cells has made it an attractive target for antineoplastic therapies.

### Brentuximab Vedotin

In clinical studies, naked anti-CD30 monoclonal antibodies (mAbs) did not reveal significant responses in patients with refractory or relapsed (R/R) cHL [15]. This is presumably related to a reduced efficiency of antibody-dependent cellular cytotoxicity (ADCC) against HRS cells due to several alterations in signaling mechanisms in these cells and in the surrounding microenvironment, which act to promote tumor growth and immune evasion as well as rapid internalization and shedding of CD30 after ligand binding [16,17,18]. However, although the rapid internalization of CD30 after ligand binding counteracts the efficient recruitment of immunological effectors involved in ADCC, it provides the ideal opportunity for utilizing anti-CD30 mAbs as a vehicle for delivering cytotoxic payloads into HRS cells.

Brentuximab vedotin (BV) is an antibody-drug conjugate (ADC) composed of an anti-CD30 mAb conjugated by a protease cleavable linker with the cytotoxic microtubule-disrupting agent monomethyl auristatin E (MMAE, vedotin). After BV binds to CD30 on the cell surface, the ADC-CD30 complex is internalized, and MMAE is released, inducing cell cycle arrest and subsequent apoptotic death. When initially tested in a phase I study conducted on 45 patients with R/R CD30-positive lymphomas, BV administered intravenously every 3 weeks showed a safe toxicity profile and interesting antitumor activity, with the majority of patients achieving an objective response [19]. Thus, in a multinational pivotal phase II trial, BV was evaluated on 102 patients with R/R cHL after ASCT: the overall response rate (ORR) was 75%, with 34% of complete remission (CR) [20]. On the basis of these results, the regulatory authorities Food and Drug Administration (FDA) and European Medicines Agency (EMA) approved the first use of BV as monotherapy for patients with R/R cHL after ASCT or at least two prior lines of therapy [21,22].

Following the first approval, BV has been evaluated in several scenarios of R/R cHL.

In patients with R/R cHL after salvage chemotherapy in a pre-ASCT setting, BV monotherapy obtained a negative positron emission tomography (PET) status in a high percentage of cases. In two multicenter retrospective analyses on ASCT-naïve patients who were PET-positive following conventional chemotherapy, salvage treatment with a median of 4 doses of BV resulted in normalization of PET scans in 8 out of 15 (53%) and in 9 out of 30 (30%) patients [23,24,25].

In the randomized, double-blind, placebo-controlled, phase III trial AETHERA, 329 patients with primary refractory or relapsed unfavorable-risk cHL were randomly assigned to receive BV or placebo for up to 16 cycles after ASCT. The median progression-free survival (PFS) was 42.9 months in the BV group and 24.1 months in the placebo group [26]. The favorable results of the AETHERA trial granted the FDA approval of BV as post-ASCT consolidation in patients with cHL at high risk of relapse or progression.

The role of BV monotherapy in R/R cHL was also analyzed in pre- and post-allogeneic stem cell transplantation (allo-SCT) settings. In a retrospective analysis, 18 patients with R/R cHL treated with BV were subsequently treated with reduced-intensity allo-SCT with successful engraftment and no unexpected toxicities [27]. Another retrospective analysis of 16 BV-naïve patients with recurrent cHL after allo-SCT who were treated with BV reported transient disease control with 69% of ORR (CR in 31%) and a median duration of response (DOR) of 5 months [28].

Few data are available about BV combined with chemotherapy in R/R cHL patients. In a phase I/II trial, the combination of BV plus bendamustine was tested as the first salvage strategy in 55 patients: ORR was 92.5%, with 73.6% of patients achieving a CR [29]. In another phase I/II, multicenter, single-arm trial, 64 patients with heavily pretreated cHL (65% previously received ASCT, allo-SCT or both) were treated with BV plus bendamustine for up to 6 cycles. Patients were excluded if they were previously treated with BV and bendamustine in combination but were eligible if they had received either as a single agent. The ORR in the combined phase I/II cohort was 71%, with a CR rate of 32% [30,31].

## 3. PD-1/PD-L1 Signaling and PD-1 Checkpoint Inhibitors

T-mediated adaptive immunity is crucial in antineoplastic response. It is initiated through antigen recognition by the T-Cell Receptor (TCR), which is regulated by a multitude of co-stimulatory and inhibitory signals. Such inhibitory signals, necessary for the maintenance of self-tolerance, are globally named Immune Checkpoints (ICs) [32,33]. One of the most studied ICs is represented by the PD-1/PD-L1 pathway, which was revealed to be an effective target for several biological drugs adopted both in hematologic malignancies and solid tumors.

PD-1 (or CD279) is a 55-kDa transmembrane receptor type I protein with canonical immunoglobulin (Ig)-like extracellular domain encoded by a gene located on chromosome 2q37. It is usually expressed by activated T cells, T regulatory cells (Tregs), T follicular helper cells, B cells, NK cells, monocytes and Dendritic Cells (DCs) [34,35]. PD-1 has two natural ligands: PD-L1, also known as CD274 or B7-H1, and PD-L2, also named as CD273 or B7-DC, encoded by two genes located on chromosome 9p24, just 42 Kb apart [36]. The two PD-1 ligands differ in their expression patterns, with PD-L1 constitutively expressed on T and B cells, monocytes, DCs, mesenchymal stem cells, bone marrow-derived mast cells and a wide range of non-hematopoietic cells, while PD-L2 expression can be induced on monocytes, DCs, bone marrow-derived mast cells and on resting peritoneal B1 cells [37]. PD-1/PD-1-ligand interaction results in clustering between the PD-1/PD-1-ligand complex and the TCR-antigen complex with the recruitment of Src Homology 2 domain-containing tyrosine Phosphatase-1 (SHP-1) and SHP-2 proteins. SHP-1 and SHP-2 are two phosphatases that dephosphorylate ZAP-70 with consequent signal attenuation downstream of TCR by inhibition of PI3K and RAS-MAPK pathways, downregulation of TNF-alpha and IL-2 production, and overall inhibition of T cells activity [38,39].

**Figure 1:**
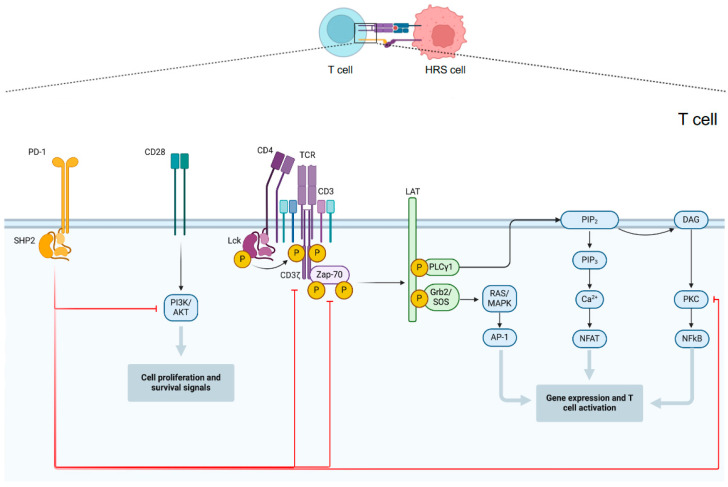
PD1-mediated inhibition of T cell activity. Multiple signal transduction pathways converge in antigen-stimulated T lymphocytes to generate transcription factors that promote the expression of genes involved in activating and maintaining the T response. The main pathways involved are those of RAS/MAPK, PLCγ1 and PI3K/AKT. PD-1 activation results in SHP2-mediated dephosphorylation of CD3ζ, Zap-70, PKC, and PI3K and consequently with their inhibition.

As previously highlighted, HRS cells represent only a small portion of the overall tumor mass and are surrounded by a variety of immune cells, yet antitumor immunity fails to effectively recognize and eliminate the malignant cells. Indeed, HRS cells achieve immune evasion by multiple mechanisms including enhanced expression of PD-L1 and PD-L2 on themselves and, through local signals, on tumor-infiltrating macrophages. PD-L1 and PD-L2 bind PD-1 on the surface of tumor-infiltrating lymphocytes suppressing T-cell activation [40]. Through PD-1 or PD-L1 targeting, immune checkpoint inhibitors unblock immune suppression of T cells and restore their antitumor activity. The mechanism of action of PD-1 inhibitors is summarized in Figure 1.

### 3.1. Nivolumab and Pembrolizumab

To date, two PD-1 inhibitors are approved for the treatment of cHL and several new compounds are under investigation.

Nivolumab is a fully human IgG4 mAb directed against PD-1 which is approved as monotherapy for patients with R/R cHL after ASCT and BV [41,42]. In a phase I trial, 23 patients with heavily pretreated cHL (most of them previously subjected to ASCT and BV) received nivolumab intravenously every 2 weeks until CR, tumor progression, or excessive toxicity. Nivolumab showed substantial therapeutic activity with an ORR of 87% (17% CR) and a good toxicity profile [43]. In an international, multicenter, multi-cohort, single-arm, phase II trial (CHECKMATE 205), 243 patients with R/R cHL after ASCT failure received nivolumab until disease progression or unacceptable toxicity. At a median follow-up of 8.6 months, the ORR was 69%, with 16% of CR [44]. The long-term follow-up analysis of the study showed durable responses with a median DOR of 18.2 months overall (33.3 months for patients achieving CR) and a 5-year overall survival (OS) of 71.4%. Interestingly, a small portion of patients who discontinued nivolumab in CR and later relapsed obtained a second response after treatment rechallenge [45]. In the CheckMate 744 phase II trial, nivolumab has also been tested as a salvage treatment for young R/R cHL patients in combination with BV +/− bendamustine in a response-adapted approach. Forty-three patients aged 5–30 years received four cycles of nivolumab plus BV, with a CR rate of 59%, while 11 patients not achieving CR underwent 2–4 further intensification cycles of BV plus bendamustine, with a global CR rate of 94%. Thirty-four patients received consolidation with ASCT. The one-year PFS rate was 91% [46].

Pembrolizumab is a humanized anti-PD-1 IgG4 mAb approved by the FDA and EMA as monotherapy for patients with R/R cHL after ASCT failure or at least two prior lines of therapy if ASCT ineligible [47,48]. In a cohort of 31 patients with heavily pretreated cHL R/R after BV and belonging to the phase Ib KEYNOTE-013 trial, pembrolizumab monotherapy administered intravenously every 2 weeks—instead of every 3 weeks as is most commonly the case—resulted in 65% of ORR with a CR rate of 16% [49]. In the multi-cohort, single-arm, phase II trial KEYNOTE-87, 210 patients with R/R cHL after ASCT and/or BV received pembrolizumab every 3 weeks for a maximum of 24 months or until disease progression, intolerable toxicity, or investigator decision. At a median follow-up of 10.1 months, the ORR was 69%, with 22.4% of patients achieving CR [50]. An updated analysis with a median follow-up of 27.6 months showed long-lasting efficacy of pembrolizumab with a median DOR of 16.5 months, a median PFS ranging from 10.9 months to not reached for patients obtaining SD to CR as the best response and a median OS not reached for all the cohorts. Of note, in this long-term analysis, slightly higher response rates were observed, with ORR and CR of 71.9% and 27.6%, respectively, showing the possibility of late responses [51].

Nivolumab and pembrolizumab efficacy was also evaluated in specific patient settings, such as after allo-SCT failure or as treatment rechallenge in patients previously exposed to anti-PD-1 drugs. In a retrospective analysis, 20 patients with cHL R/R after allo-SCT received nivolumab, which resulted in effective and relatively safe in this setting. The ORR was 95% with 42% of CR and 1-year PFS and OS of 58.2% and 78.7%, respectively. Six patients (30%) experienced graft versus host disease after nivolumab initiation, and two of them died due to this condition [52]. As part of the KEYNOTE-87 trial, 10 patients with relapsing cHL after 12 months since the last pembrolizumab infusion were treated with a second course of pembrolizumab. The ORR was 75% with 50% of patients achieving a second CR [51].

### 3.2. Other PD-1 Checkpoint Inhibitors

Sintilimab and tislelizumab are two novel humanized IgG4 mAbs directed against PD-1 developed and approved in China for the treatment of R/R cHL. In two multicenter, single-arm, phase II studies (ORIENT-1 and BGB-A317-203), more than 170 patients with R/R cHL after ≥2 lines of therapy received sintilimab 200 mg flat dose or tislelizumab 200 mg flat dose every 3 weeks until progression, unacceptable toxicity, or for a maximum of 2 years. The ORR obtained was up to 80%, with CR rates ranging from 30 to 60% [53,54].

GLS-010 (zimberelimab) is another novel, fully human, IgG4 mAb with high affinity and selectivity for PD-1. In a multicenter, single-arm, phase II trial, 85 patients with R/R cHL after at least two prior lines of therapy received GLS-010 240 mg flat dose every 2 weeks until confirmed disease progression or up to a maximum of 2 years. The ORR was 90.6%, with a CR in 32.9% of patients [55].

Avelumab is a fully human, anti PD-1 IgG1 mAb. It inhibits PD-1/PD-1L interaction and, unlike the mAbs described so far, it is also able to determine ADCC activation against HRS cells due to its different Fc portion. In a multicenter, multiple-dose, phase I trial (JAVELIN), 31 patients with heavily pretreated R/R cHL was randomized to receive avelumab at different dosages. The ORR was 41.9%, with a CR rate of 19.4% [56].

### 3.3. Chemotherapy-Resensitization after PD-1 Inhibition

Retrospective analyses on solid tumors and cHL revealed an improvement in response rates to chemotherapy after exposure to anti-PD-1 mAbs. In three retrospective small series, patients with heavily pretreated cHL who showed unsatisfactory responses to PD-1 inhibitors were treated with different chemotherapy strategies with or without concomitant anti-PD-1 administration. Around 60–68% of patients obtained a sustained objective response, with up to 40% of them achieving a CR and a median PFS ranging from 8 to 11 months among the different cohorts. A portion of the responding patients underwent ASCT or allo-SCT without excess toxicities [57,58,59]. Interestingly, in the series reported by Rossi et al. [57] 12 out of 15 patients responded to the same chemotherapy regimen to which they previously resulted refractory. These results led to the hypothesis that exposure to anti-PD-1 mAbs could resensitize tumor cells to chemotherapy, further encouraging their use in multi-refractory patients.

## 4. Frontline Treatment Reshaping

### 4.1. BV Plus Chemotherapy Combinations

Advanced stage cHL is usually treated with chemotherapy, and as previously mentioned, the most common schemes used in this setting are ABVD and eBEACOPP [2,3]. Nevertheless, in the last decade, BV, in combination with different chemotherapy regimens, has been established as an effective first-line alternative in advanced-stage cHL.

The ECHELON-1 trial led to the approval of the combination of BV plus doxorubicin, vinblastine, and dacarbazine (BV-AVD) as the first-line treatment of advanced-stage cHL [21,60]. The 5-year update of this international randomized phase III study showed a PFS benefit of BV-AVD over the ABVD standard arm (5-year PFS of 82.2% vs. 75.3%, HR 0.69, *p* = 0.0017) [61]. The benefits of BV-AVD were consistent in different subgroups of patients regardless of treatment response at the interim-PET (PET-2 status), disease stage and other prognostic factors, and they were also confirmed by real-world studies [61,62,63,64]. Moreover, the last 6-year long-term analysis of the trial demonstrated an improved OS for BV-AVD in respect of the standard arm (93.9% and 89.4%, respectively, HR 0.68), thus confirming the higher effectiveness of the immunochemotherapy combination in respect of standard chemotherapy for treatment-naïve advanced stage cHL [65]. Despite the improved efficacy of BV-AVD, this combination showed increased toxicities compared to ABVD in terms of febrile neutropenia and peripheral neuropathy rates (47–80% with 8–10% grade 3) even if fewer secondary malignancies have been reported [61,65,66].

BV combined with chemotherapy has also been tested in patients with limited-stage disease and other subgroups of cHL patients. In the multicenter randomized phase II trial BREACH, BV-AVD has been investigated in early-stage unfavorable cHL. Patients treated with four courses of BV-AVD followed by 30 Gy of involved nodal radiotherapy (INRT) showed higher PET-2 negative rates and better PFS with respect to the standard 4 ABVD + INRT [66]. Abramson et al. [67] treated non-bulky stage I-II cHL with BV plus doxorubicin and dacarbazine (BV-AD), obtaining ORR and CR rates of 100% and 97%, respectively, with an estimated 5-year PFS of 91%. Among treated patients, 92% achieved a CR at PET-2 and received four cycles, while the remaining patients received six total cycles. Kumar et al. [68] confirmed the high efficacy of four cycles of BV-AVD in early stages and unfavorable risk cHL, even omitting radiotherapy in PET-4 negative patients (2-year PFS 96.6%).

BV-AVD has been shown to be safe and effective also in advanced-stage HIV-related cHL [69]. In a phase I–II trial conducted on 41 HIV-positive patients, 90% of them achieved a CR, and the 2-year PFS was 87% without excess toxicities observed [70].

BV was also tested as a frontline therapy in older or unfit patients to reduce or avoid chemotherapy exposure. While BV monotherapy granted a high ORR but limited PFS and OS [71,72,73], the addition of reduced-intensity chemotherapy showed promising results. Evens et al. [74] tested a sequential treatment modality with single-agent BV administered before and after AVD in previously untreated patients aged 60 or older. At the end of the AVD cycles, the response rates were remarkable, with 96% of patients achieving a CR. The study showed considerably higher outcomes with respect to those reported in the last few decades in this age group, with 2-year OS and PFS of 93% and 84%, respectively, alongside a manageable toxicity profile. In another two-arm non-randomized phase II study on older patients with cHL non-eligible for standard chemotherapy, BV plus dacarbazine granted 62% of CR with good tolerability. Patients enrolled in the second arm of the study received BV plus bendamustine with higher CR rates (88%) at the cost of a greater rate of adverse events and lower tolerability [75,76].

Eichenauer et al. [77] included BV in the higher intensity scheme eBEACOPP in a phase II study with the aim of lowering chemotherapy-related toxicities and maintaining sufficient efficacy. Patients were randomized to receive either a combination of BV, etoposide, doxorubicin, cyclophosphamide, dacarbazine, and dexamethasone (BrECADD) or BV, etoposide, doxorubicin, procarbazine, and prednisone (BrECAPP) as eBEACOPP variants. Both schemes granted satisfactory responses at the end of treatment compared to the usual eBEACOPP performance. The estimated 18-month PFS was 95% for BrECAPP and 89% for BrECADD. In both arms, fewer dose reductions than eBEACOPP were necessary, and the toxicity profile was especially favorable for BrECADD. Interestingly, the incidence of peripheral neuropathy was 32–35%, which is lower than what has been observed with other schemes containing BV. Recently, preliminary data about the phase III HD21 trial comparing standard eBEACOPP and BrECADD in advanced-stage cHL were shown by the German Hodgkin Study Group (GHSG). With a median follow-up of 40 months, the 3-year PFS was 92.3% for eBEACOPP and 94.9% for BrECADD. The 3-year OS was 98.5% in both groups [78].

### 4.2. PD-1 Checkpoint Inhibitors plus Chemotherapy Combinations

PD-1 inhibitors are not currently approved for frontline treatment, but a number of trials have shown their promising efficacy and limited toxicity when combined with chemotherapy, especially in limited-stage disease.

The NIVAHL trial investigated the combination of nivolumab plus AVD (N-AVD) in early-stage cHL patients with unfavorable disease by GHSG criteria, with impressive efficacy results. Patients were randomized to receive four courses of N-AVD or a sequential treatment consisting of 4 doses of nivolumab, two cycles of N-AVD, and two cycles of AVD and patients in both arms underwent radiation therapy afterward [79]. The 3-year follow-up analysis of this phase II study showed 3-year PFS and OS estimates of 99% and 100%, respectively [80]. Fifteen percent of patients required interventions for potentially treatment-associated morbidities, though none required immunosuppressants. The most common adverse events (AEs) were hypothyroidism and respiratory disorders, which were documented in 19% of patients.

The phase II study CheckMate 205, conducted on advanced-stage cHL patients, tested a sequential treatment scheme consisting of four doses of nivolumab followed by six cycles of N-AVD, reaching high response rates (80% CR) with a reasonable toxicity profile [81]. Rutherford et al. [82] compared frontline BV-AVD and N-AVD in advanced stage cHL in a head-to-head phase III trial. Although hypothyroidism and cutaneous rashes were more often observed with N-AVD, this scheme resulted in generally less toxicity and better tolerance, with a discontinuation rate of 15% vs. 39% in patients treated with BV-AVD. Even though only a limited follow-up is available, N-AVD was associated with improved PFS and OS over BV-AVD, with 1-year PFS of 93% vs. 64% and 1-year OS of 95% vs. 83%.

Pembrolizumab has been tested in a frontline therapy setting as well. Allen et al. [83,84] tested sequential pembrolizumab for three cycles followed by 4 to 6 cycles of AVD in unfavorable or advanced-stage cHL, reaching an astounding CR rate of 100%. None of the patients discontinued the treatment, and all of them were alive and disease-free at the last update. Similar results have been obtained by Lynch et al. [85] using a combination of pembrolizumab and AVD (P-AVD) instead of sequential therapy. This scheme granted a CR rate of 90% with 2-year PFS and OS of 97% and 100%, respectively. Of note, among patients with persistent PET positivity at the end of treatment, none had relapsed at the time of publication, suggesting the possibility of spurious PET findings with P-AVD.

### 4.3. BV-Nivolumab Combination and Chemo-Free Regimens

Given the high efficacy and limited toxicity of BV and PD-1 inhibitors, the combination of these compounds could represent a valid alternative to conventional chemotherapy for patients ineligible for standard schemes due to age or comorbidities. The ACCRU trial tested eight cycles of BV plus nivolumab (BV-N) in patients ineligible for chemotherapy. The results were encouraging, with an ORR of 61% and a CR rate of 48%, maintaining good tolerance throughout the full treatment [86]. Friedberg et al. [87] conducted a non-comparative phase II study on cHL patients aged ≥60 years unfit for conventional chemotherapy, testing two combinations: BV plus dacarbazine (BV-DTIC) or BV-N. With a median follow-up of around 5 years, upfront BV-N obtained an ORR of 86% with median PFS and OS not reached, while BV-DTIC granted a median PFS of 47.2 months. Despite the advanced age and significant frailty of enrolled patients, both regimens showed good tolerance and long-term efficacy, thus suggesting potential alternatives to chemotherapy in this setting. Notably, the chemo-free regimen had more G3-4 AEs in respect of BV-DTIC (76% vs. 45%).

The combination of standard chemotherapy and novel compounds has also granted interesting results in limited-stage cHL. Park et al. [88] tested a new PET-driven treatment scheme in patients with limited-stage, non-bulky cHL composed of three cycles of BV-AVD followed by up to eight cycles of nivolumab. Patients who were still PET-positive at the end of the three BV-AVD cycles received an additional four cycles of BV-N before nivolumab consolidation. The results are astonishing, with 100% of PFS at 22 months, although a longer follow-up is needed to establish the true benefit of this approach in limited-stage cHL. In the ongoing SGN35-027 trial, patients with limited-stage cHL receive nivolumab in addition to BV-AD. Early results showed an ORR of 95–98% with 88–93% of CR and a 1-year PFS of 100% [89,90].

Currently ongoing phase II–III evaluating new regimens for the frontline treatment of adult patients with cHL are summarized in Table 1.

## 5. Regulation of T-Lymphocytes and NK Cells Function

### 5.1. CD25 Targeting

Camidanlumab tesirine (cami-T) is an anti-CD25 ADC comprised of an IgG1 directed against CD25 and the cellular toxin pyrrolobenzodiazepine (tesirine). CD25, or IL2RA, is an important receptor expressed by circulating activated immune cells and Tregs as well as by many hematological malignancies, including cHL [91]. Tregs play an important role in the persistence of cancers: an excessive infiltration of Tregs into the TME can imbalance the Tregs/T-effector cells ratio, contributing to tumor progression [92]. Cami-T showed antitumor activity in vivo via different mechanisms, including direct DNA toxicity in neoplastic cells as well as through depletion of CD25-positive Tregs [93]. Interestingly, CD25 tumor expression levels did not relate to response to this compound. On the other hand, lower levels of circulating CD25-positive cells might correlate with poor responses [94].

A phase I study was conducted on patients affected by various types of R/R lymphoproliferative disorders, including cHL. In this setting, cami-T achieved an ORR of above 70% for cHL patients [95]. The phase II study by Herrera et al. [96] showed similar results, with ORR between 66.2% and 78.4% among different subgroups of patients. A phase II trial enrolling 117 multi-refractory cHL patients previously exposed to both BV and anti-PD-1 confirmed the encouraging efficacy of cami-T in this challenging setting. The ORR was 70.1%, with 33.3% of CR and a median DOR of 13.7 months for all responders [97]. While cami-T showed a moderate incidence of both hematological and non-hematological adverse events with anemia, pyrexia, skin reactions and isolated gamma-glutamyl transferase elevation being the most common [95,98], concern has grown about its autoimmune toxicities, possibly caused by the depletion of CD25-positive Tregs. Indeed, in the phase II trial, Carlo-Stella et al. reported eight cases (6.8%) of Guillain–Barré syndrome/polyradiculopathy, half of them without recovery at data cutoff [97].

Besides the antitumor activity of cami-T employed as a single agent, preclinical in vitro and in vivo studies showed interesting synergistic activity in combination with gemcitabine [99].

### 5.2. Other Immune Checkpoint Pathways and Novel Checkpoint Inhibitors

**Figure 2:**
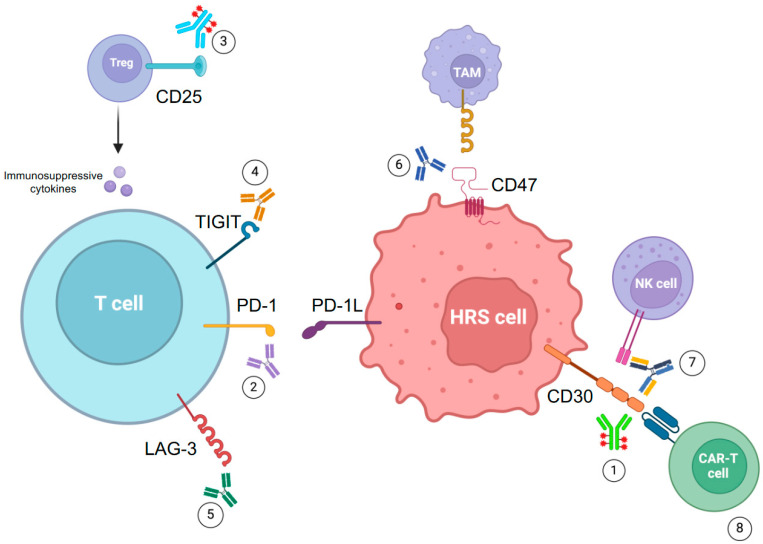
Current target therapy approaches in cHL. 1: Brentuximab vedotin. 2: Nivolumab, pembrolizumab and other PD-1 inhibitors. 3: Camidanlumab tesirine. 4: Vibostolimab. 5: Favezelimab. 6: Magrolimab. 7: AFM13. 8: anti-CD30 CAR T-cells.

Several IC pathways have been studied besides PD-1/PD-1L, with the aim of developing new drugs to determine better immune activity against HRS cells (Figure 2).

#### 5.2.1. TIGIT Blockade

Among immune checkpoints, TIGIT (T-cell immunoreceptor with immunoglobulin and immunoreceptor tyrosine-based inhibitory motif domains) is an inhibitor receptor present on immune cells, including T and NK cells, that is activated by different ligands that can be expressed by neoplastic cells such as CD155 and CD112. TIGIT can be targeted by selective compounds such as the anti-TIGIT humanized mAb vibostolimab, resulting in effective antitumor responses against both solid tumors and hematological neoplasms [100]. Anti-TIGIT and anti-PD-1 drugs have shown in vitro and in vivo synergic action [101]. A phase II study aimed to evaluate the safety and efficacy of the combination pembrolizumab–vibostolimab in patients with cHL, NHLs and multiple myeloma is ongoing [102].

#### 5.2.2. LAG-3 Targeting

Lymphocyte-activation gene 3 (LAG-3) regulates T cell function and has a major function in many tumors, including cHL. LAG-3 is heavily expressed in exhausted T cells, especially in the context of persistent antigenic stimulation [103]. In vitro studies showed the synergistic activity of LAG-3 and PD-1 in suppressing the immune response against neoplastic cells [104].

Favezelimab is an anti-LAG-3 mAb that is currently being tested in combination with pembrolizumab in R/R cHL and other hematological malignancies [105,106]. Johnson et al. [106] showed that the combination of favezelimab and pembrolizumab administered to anti-PD-1-naïve patients granted an ORR and a CR rate of 80% and 33%, respectively, with a median PFS of 19.4 months and a 2-year OS of 93%. The toxicity profile was manageable, with the most common AEs consisting of hypothyroidism, infusion-related reactions, fatigue and nausea. Results of the phase I study by Timmerman et al. [105], conducted on 34 anti-PD-1 refractory patients, were recently updated: an ORR of 29% and a CR rate of 9% were reported, showing a sizeable efficacy of the combined therapy even in patients previously exposed to anti-PD-1 drugs. A phase III study from Lavie et al. [107], which aims to compare the combination of favezelimab and pembrolizumab vs. chemotherapy in a similar population of anti-PD-1 pretreated patients, is ongoing.

#### 5.2.3. CD47 Blockade

The CD47-SIRPα pathway is a “do not eat me” signal expressed by several cancers, including cHL, which leads to the avoidance of phagocytosis through interaction with Signal-Regulatory Protein alpha (SIRPα) expressed on TAMs and other phagocytes [108,109,110]. In cHL, CD47 intensity of expression is variable, and higher levels of CD47 expression correlate with inferior outcomes [111,112]. Hu5F9-G4 (magrolimab), a novel humanized IgG4 anti-CD47 mAb, has shown moderate efficacy in the treatment of acute myeloid leukemia and myelodysplastic syndromes in combination with azacitidine and in the treatment of NHLs in combination with rituximab [113,114,115]. Based on these data and on the key role of the “Hodgkinian” TME in the pathogenesis and immune escape of cHL, an ongoing phase II trial is evaluating the combination of pembrolizumab plus magrolimab in patients with R/R cHL after ≥2 lines of therapy (NCT04788043).

### 5.3. NK Targeting and Activation: CD30 × CD16 Bispecific Antibodies

AFM13 is a tetravalent bispecific antibody (bsAb) that binds CD30 and CD16A—an isoform of CD16 mainly expressed on NK cells and macrophages—with high affinity and specificity [116,117]. Through this, CD16 and CD30 double-binding AFM13 showed consistent antitumor activity, provoking directed polyfunctional activation of mature NK cells against CD30-expressing neoplastic cells [116,118]. AFM13 infusion results in a transient dose-independent decrease in circulating NK cells and a relative increase in activated NK cells [119]. Moreover, AFM13 binding not only activates NK cells but enhances their cytotoxic effect on neoplastic cells as well as their sensibility to activating cytokines such as IL2 and IL15 [117]. While both immature and mature NK cells are activated by AFM13, NK cell responses are quite heterogeneous depending on the recruited NK cell population: there is evidence that NK cells of heavily pretreated patients show lower cytotoxic activity compared to those of healthy subjects [116].

As a single agent, AFM13 revealed a favorable toxicity profile but scant antitumor activity. Rothe et al. [119] conducted a phase I study on 28 heavily pretreated cHL patients, showing low rates of G ≥ 3 AEs (9.2%), the most common being pyrexia, chills, headache and a single dose-limiting toxicity event characterized by hemolytic anemia. However, ORR was 11.5%, although no CR was observed. Similar results were seen in a multicenter phase II trial by Sasse et al. [120], which achieved an ORR of 16.7% and only two serious AEs.

Bartlett et al. [121] combined AFM13 and pembrolizumab in R/R cHL, greatly increasing efficacy: an ORR of 83% has been achieved, with 37% of patients obtaining a CR. The mean DOR was 9.9 months. Moreover, this trial showed that also patients previously treated with BV could achieve a response with AFM13. Indeed, there is evidence that BV does not diminish CD30 expression in refractory patients [121,122].

AFM13 has also been tested in combination with different cell products with promising results [118,123]. In the combination of AFM13 with pre-activated and expanded cord-blood-derived NK cells in patients with double-refractory cHL, the toxicity profile was manageable with high response rates (ORR 92.8%, CR 66.7%) and median event-free survival (EFS) and OS of 8 months and not reached, respectively, after a median follow-up of 14 months [124].

Of note, AFM13 efficacy could be limited by the development of anti-drug antibodies, which have been detected in 53% of patients enrolled in the phase I study by Rothe et al. [119].

## 6. Epigenetic Modulation

Disruption of normal epigenetic regulation of gene expression is a hallmark of cancer and contributes to human tumor development and progression. As epigenetic changes are reversible, they represent a potential target for cancer therapy [125].

### 6.1. DNA Methyltransferase Inhibition

DNA methylation by DNA methyltransferase enzymes (DNMT) typically represses gene transcription. Thus, DNA hypermethylation may reduce the expression of key tumor suppressor genes in human cancers, and tumor cells in cHL have been found to be characterized by a high number of hypermethylated genes [125,126]. DNMT inhibitors (DNMTi) such as azacitidine and decitabine could reverse this aberrant epigenetic regulation and have already been shown to be effective on myelodysplastic syndromes and acute myeloid leukemia [127,128]. In a case report, the use of azacitidine in a patient concurrently affected by cHL and a therapy-related myelodysplastic syndrome showed a reduction in both cHL tumor burden and metabolic activity [129].

Moreover, it is thought that DNMTi could upregulate immune signaling, priming the immune system and thus enhancing the efficacy of concomitant or subsequent therapy with immune checkpoint inhibitors [130,131]. In a phase II trial enrolling 61 R/R cHL patients, those who received the anti-PD-1 camrelizumab plus decitabine had higher rates of CR and better PFS in respect of those who received camrelizumab alone (CR 79% vs. 32%; median PFS 35 months vs. 15.5 months, respectively) [132]. Other preliminary data suggest that hypomethylating agents could restore cancer sensitivity to immune checkpoint inhibitors: 19 patients with cHL refractory to pembrolizumab and/or nivolumab were treated with CC-486 (an oral hypomethylating agent) and nivolumab. ORR was 63%, with a 10% CR rate and an estimated median PFS of 11.3 months [133].

### 6.2. Histone Deacetylase Inhibition

Epigenetic modification of histone proteins controls chromatin structure and, thus, gene expression. Histone deacetylases (HDACs) are enzymes that negatively regulate gene expression by removing acetyl groups from histones, causing chromatin condensation [125]. In cHL, overexpression of some specific isoforms of HDACs appears to correlate with shorter survival [134]. HDAC inhibitors (HDACi) have thus been tested as monotherapy or combined with other drugs in early-phase clinical trials with evidence of potential therapeutic efficacy in heavily pretreated patients with cHL. In a large trial involving 129 patients, the pan-HDACi panobinostat administered three times per week at the dose of 40 mg showed an ORR of 27%, with a median DOR of 6.9 months. Of interest, five patients achieved a CR, with a DOR of up to 15 months. Most common grade ≥ 3 AEs were related to hematological toxicity, with thrombocytopenia occurring in up to 79% of patients [135]. The HDACi vorinostat demonstrated disappointing efficacy when tested as monotherapy on R/R cHL patients [136]. Nevertheless, its combination with mTOR inhibitors such as everolimus or sirolimus showed promising results in a cohort of 40 patients, with an ORR of 45%. The median PFS of the whole cohort was less than 6 months. However, 2 of the 6 patients treated with vorinostat and sirolimus who achieved a CR remained on treatment for more than 3 and a half years. Toxicities were manageable, with hematological toxicities being the grade ≥ 3 AEs most commonly encountered [137].

Of interest, a phase II clinical trial combining two epigenetic modulating agents—the HDACi tucidinostat and the DNMTi decitabine—and the immune checkpoint inhibitor camrelizumab is currently recruiting in China (NCT04233294).

### 6.3. Other Epigenetic Modulating Agents

Histone methylation promoted by histone methyltransferase EZH2 results in the silencing of genes associated with cell differentiation. Recurrent gain of function mutations of this gene has been found in many kinds of cancer, including cHL [126,138]. A phase I trial tested the EZH2 inhibitor SHR2554 on patients with R/R mature lymphoid neoplasms, including B-NHLs, T-NHLs and cHL, showing a favorable toxicity profile and promising treatment efficacy (ORR 46%) with a response achieved in 4 out of 21 patients with cHL (19%) [139].

## 7. JAK/STAT Blockade

The JAK/STAT signaling pathway, which plays a key role in promoting physiological cell proliferation and survival, has been shown to be commonly dysregulated in cHL [140]. In an analysis of biopsy samples from 30 patients with cHL, Tiacci et al. [141] showed that 87% of cases carried genetic alterations in multiple genes involved in the JAK/STAT pathway. Different mechanisms have been observed, including JAK2 overexpression secondary to 9p24.1 genomic amplification, mutations in other genes of the pathway, such as JAK1, STAT3, STAT5B or STAT6, or inactivating mutations in negative regulators, such as SOCS-1 [141,142]. Moreover, in vitro experiments suggest that pharmacological blockade of the JAK/STAT pathway has a direct cytotoxic effect on malignant cells and helps in regulating the pro-inflammatory TME [143].

The afore-mentioned observations led the way to human clinical trials. Ruxolitinib, a selective inhibitor of JAK1 and JAK2, which is already approved for the treatment of myeloproliferative neoplasms, has been the most widely employed molecule against cHL, showing good tolerability but overall modest efficacy. Kim et al. [144] described the effect of the administration of ruxolitinib 20 mg twice daily to 13 patients with R/R cHL: the disease control rate was 54%, and the median DOR was 5.6 months. One patient achieved a CR, which was maintained for 15 28-day cycles. Grade 3 AEs were restricted to anemia and neutropenia, affecting a total of three patients, all of whom recovered without the need for dose reduction. Of the 33 patients with R/R cHL receiving ruxolitinib in the phase II trial conducted by Van Den Neste et al. [145], only 6 (18.8%) achieved a response, with a median DOR of 7.7 months and a median PFS of 3.5 months. Gillessen et al. [146] reported the results of a phase II trial with ruxolitinib administered at the dose of 25 mg twice daily in patients with R/R cHL after at least two lines of therapy. ORR after two 28-day cycles was 16.7%, with a median PFS of 3.6 months and an estimated 1-year OS of 50.6%. The trial was stopped early due to the low response rate observed.

While the inhibition of the JAK/STAT pathway alone seems to lead to transient responses only, the good tolerability profile of ruxolitinib monotherapy led the way to the investigation of combination therapies. Bachanova et al. [147] reported the results of the combination of ruxolitinib plus nivolumab tested in 19 patients with R/R cHL who had already failed prior therapy with another PD-1 checkpoint inhibitor. At a median follow-up of 13 months, the ORR was 75%, and the 1-year PFS was 64%. The capability of ruxolitinib to reshape the TME and reduce the production of immunosuppressive cytokines may explain the promising efficacy of the combination, even in patients who already failed a treatment with anti-PD-1 [143].

The association of itacitinib, a selective JAK1 inhibitor, with everolimus has also been studied. In 14 heavily pre-treated cHL patients who had no other treatment options available, the ORR was 79%, and at a median follow-up of 6.8 months, the median PFS was estimated to be 3.8 months [148].

## 8. Chimeric Antigen Receptor T-Cell Therapy

In the last decade, anti-CD19 CAR T-cells have revolutionized the therapeutic paradigm of many B-NHLs and B-cell acute lymphoblastic leukemia. Autologous lymphocytes are collected and engineered with the transduction of a CAR by a replicant-incompetent viral vector and are then reinfused to the patient, causing the killing of tumor cells through T-cell recognition of a specific antigen [149].

CAR T-cell constructs targeting the CD30 antigen have been tested in cHL in phase I and phase II trials revealing to be effective and safe, with low incidence of the typical CAR T-cells-related toxicities cytokine release syndrome (CRS) and immune-effector cell-associated neurotoxicity syndrome (ICANS) (Figure 2) [150].

In the first published phase I trial of an anti-CD30 CAR T-cell product, 17 patients with R/R cHL and one patient with anaplastic large cell lymphoma were treated, showing 39% of ORR and a median PFS of 6 months [151]. In a phase I/II trial published by Ramos et al. [152], 41 patients with heavily pre-treated cHL (median of 7 prior lines of therapy including BV, an anti-PD-1 mAb and ASCT or allo-SCT) received an anti-CD30 CAR T-cell product: the ORR of the whole cohort was 62% with a 1-year PFS of 36%. Among the 32 patients who received a fludarabine-based lymphodepletion before CAR T-cells infusion, ORR was 72%, with 91 patients (59%) achieving a CR; the 1-year PFS and OS of this subgroup resulted in 61% and 94%, respectively. Of note, no neurologic toxicity was observed, and all instances of CRS were low-grade. These favorable results were confirmed in a phase II study on 15 multi-refractory cHL patients in which anti-CD30 CAR T-cell infusion resulted in an ORR of 73.3% with a CR rate of 60% and very low rates of acute toxicities. Seven patients received a second infusion, which induced further responses: all five evaluable patients achieved a response [153].

Despite high response rates, disease progression after anti-CD30 CAR T-cells is frequent, and treatment failures have been mainly correlated to a high value of metabolic tumor volume by PET scan performed before CAR T-cell infusion [154]. In patients relapsing after CAR T-cell treatment, PD-1 inhibitors could be of some effect in re-inducing a response, even in patients previously exposed or progressed under these drugs. Voorhees et al. [155] described the outcomes of 10 patients treated with PD-1 inhibitors after anti-CD30 CAR T-cell treatment failure. Of note, 7 out of 10 had prior anti-PD-1 exposure. All 10 patients had an objective response, with 7 achieving a CR. With a median follow-up of 3.6 years, median PFS was not reached, and most patients remained in ongoing response. Expansion and/or persistence of circulating CAR T-cells following anti-PD-1 therapy was observed in the blood samples of all three patients with longitudinal blood samples available. An upfront combination of CAR T-cells with checkpoint inhibitors could be an intriguing strategy to explore. In a small trial, 10 patients with R/R cHL were infused with expanded ex-vivo autologous or allogenic T-cells recognizing tumor-associated antigens: concomitant nivolumab administration was associated with increased persistence of these T-cell populations, suggesting a benefit of the combination [156].

Despite HRS cells’ lack of CD19 expression, it has been hypothesized that also anti-CD19 CAR T-cells could have a certain antitumor activity in cHL due to targeting of the non-malignant B-cells included in the TME and of the CD19-positive clonotypic B-cells thought to be responsible for the continued generation of HRS cells [157,158]. A small pilot study appeared to confirm the hypothesis: among four heavily pretreated patients with cHL infused with a non-viral RNA anti-CD19 CAR T-cell construct, one achieved a CR and another a PR [159]. These observations could lead the way to the development of CAR T-cell therapies targeting multiple antigens at once in cHL.

Currently ongoing trials testing CAR T-cell products and other novel agents in adult patients with R/R cHL are summarized in Table 2.

## 9. Conclusions

Despite cHL being a highly curable disease, the treatment approach for R/R patients results in challenges for physicians. However, in the last decades, the increasing understanding of cHL biology and its mechanisms of treatment resistance led to relevant steps forward in curacy rates of the disease, with the development of many new treatment options.

The introduction of BV and PD-1 checkpoint inhibitors represented a treatment paradigm shift for R/R cHL. More recently, the BV-AVD combined modality became the new frontline standard of care for advanced-stage cHL patients, and upcoming final results from the BrECADD scheme and the upfront adoption of nivolumab in the N-AVD regimen will probably further improve the therapeutic landscape for treatment-naïve patients. Anti-CD30 CAR T-cells represent maybe the most promising strategy for multirefractory patients although their efficacy is still short-lasting, and a number of studies aimed to improve the T-cell antitumor performance and overcome this limitation are ongoing. Several novel compounds are under investigation aimed to enhance the antitumor immune-activity through T-cell and NK-cell modulation, such as new checkpoint inhibitors, the CD16xCD30—NK-engaging bispecific antibody AFM13 and the anti-CD25 ADC Cami-T, revealing promising efficacy in the R/R cHL setting. Lastly, targeting epigenetic modifications in R/R cHL could be a valuable therapeutic strategy, and the immunomodulating effects of epigenetic modulators hold the basis for combination therapies with high efficacy.

All the ongoing studies on novel drugs, together with the expanding knowledge of the biological complexity of cHL, will hopefully improve the curability of this disease in the future.

## Figures and Tables

**Table 1:** Currently ongoing phase II–III studies for frontline treatment of adult patients with cHL.

Title	Identifier	Phase	Regimen
Nivolumab and Brentuximab Vedotin in Treating Older Patients With Untreated Hodgkin Lymphoma	NCT02758717	Phase II	BV + N
PET Adapted Brentuximab Vedotin and Pembrolizumab in Combination With Doxorubicin and Dacarbazine in Classic Hodgkin Lymphoma	NCT05922904	Phase II	P + BV + AD
A Frontline Therapy Trial in Participants With Advanced Classical Hodgkin Lymphoma	NCT01712490	Phase III	BV + AVD vs. ABVD
Clinical Trial of Brentuximab Vedotin in Classical Hodgkin Lymphoma	NCT03646123	Phase II	BV + AVD or BV + N + VD
Fitness-adapted, Pembrolizumab-based Therapy for Untreated Classical Hodgkin Lymphoma Patients 60 Years of Age and Above	NCT05404945	Phase II	P + AVD vs. P + BV
Brentuximab Vedotin and Combination Chemotherapy in Treating Patients With Stage II–IV HIV-Associated Hodgkin Lymphoma	NCT01771107	Phase I–II	BV + AVD
Brentuximab Vedotin in Early Stage Hodgkin Lymphoma (RADAR)	NCT04685616	Phase III	BV + AVD vs. PET adapted ABVD ± RT
Doxorubicin, Vinblastine, Dacarbazine, Brentuximab Vedotin, and Nivolumab in Treating Patients With Stage I-II Hodgkin Lymphoma	NCT03233347	Phase II	BV + N + AVD
Very Early PET-response Adapted Targeted Therapy for Advanced Hodgkin Lymphoma: a Single-Arm Phase II Study (COBRA)	NCT03517137	Phase II	BV + AVD ± BrECADD
HD21 for Advanced Stages	NCT02661503	Phase III	BrECADD vs. BEACOPP
Immunotherapy (Nivolumab or Brentuximab Vedotin) Plus Combination Chemotherapy in Treating Patients With Newly Diagnosed Stage III–IV Classic Hodgkin Lymphoma	NCT03907488	Phase III	BV-AVD vs. N-AVD
A Study to Compare Standard Therapy to Treat Hodgkin Lymphoma to the Use of Two Drugs, Brentuximab Vedotin and Nivolumab	NCT05675410	Phase III	BV and N + standard chemotherapy
A(B)VD Followed by Nivolumab as Frontline Therapy for Higher Risk Patients With Classical Hodgkin Lymphoma (HL)	NCT03033914	Phase I–II	ABVD + N
Lowdose Nivolumab in Combination With AVD as Front Line Therapy for Classic Hodgkin’s Lymphoma	NCT05772624	Phase II	AVD + Low dose N
Pembrolizumab in First-Line Treatment of Advanced-Stage Classical Hodgkin Lymphoma (Pembro-FLASH)	NCT06045195	Phase II	P-EACOPP
A Phase II Study to Determine Pembrolizumab as Frontline Treatment of Patients With Hodgkin Lymphoma (PLIMATH)	NCT03331731	Phase II	P
Fitness-adapted, Pembrolizumab-based Therapy for Untreated Classical Hodgkin Lymphoma Patients 60 Years of Age and Above	NCT05404945	Phase II	P + AVD vs. BV + P
Study of Pembrolizumab With Bendamustine in Hodgkin Lymphoma	NCT04510636	Phase II	P + Bendamustine
Study of Safety and Efficacy of Pembrolizumab and Chemotherapy in Participants With Newly Diagnosed Classical Hodgkin Lymphoma (cHL) (MK-3475-C11/KEYNOTE-C11)	NCT05008224	Phase II	Sequential P + chemotherapy
Doxorubicin Hydrochloride, Pembrolizumab, Vinblastine, and Dacarbazine in Treating Patients With Classical Hodgkin Lymphoma	NCT03331341	Phase II	P + AVD
Pembrolizumab Followed by Chemotherapy for the Treatment of Patients With Classical Hodgkin Lymphoma	NCT06164275	Phase II	P + AVD
PET-Directed Therapy With Pembrolizumab and Combination Chemotherapy in Treating Patients With Previously Untreated Classical Hodgkin Lymphoma	NCT03226249	Phase II	P + AVD

ABVD (Adriamycin, Bleomycin, Vinblastine, Dacarbazine), AVD (Adriamycin, Vinblastine, Dacarbazine), AD (Adriamycin, Dacarbazine), BEACOPP (Bleomycin, Vincristine, Procarbazine, Prednisone, Etoposide, Doxorubicin, Cyclophosphamide), BrECADD (Brentuximab Vedotin, Etoposide, Cyclophosphamide, Doxorubicin, Dacarbazine, Dexamethasone), BV (Brentuximab Vedotin), EACOPP (Vincristine, Procarbazine, Prednisone, Etoposide, Doxorubicin, Cyclophosphamide), N (Nivolumab), P (Pembrolizumab), RT (Radiotherapy), VD (Vinblastine, Dacarbazine).

**Table 2:** Currently ongoing trials including novel agents in adult patients with R/R cHL.

Title	Identifier	Phase	Regimen	Mechanism of Action
A Study of Coformulated Favezelimab/Pembrolizumab (MK-4280A) Versus Physician’s Choice Chemotherapy in PD-(L)1-refractory, Relapsed or Refractory Classical Hodgkin Lymphoma (MK-4280A-008)	NCT05508867	Phase III	Favezelimab + Pembrolizumab vs. Physician’s choice	Anti-LAG-3 + anti-PD1
Study to Evaluate the Safety and Efficacy of a Combination of Favezelimab (MK-4280) and Pembrolizumab (MK-3475) in Participants With Hematologic Malignancies (MK-4280-003)	NCT03598608	Phase I–II	Favezelimab + Pembrolizumab	Anti-LAG-3 + anti-PD1
Phase 2 Study of AFM13 in Combination With AB-101 in Subjects With R/R HL and CD30+ PTCL	NCT05883449	Phase II	AFM13 + AB101	CD30xCD16a bsAbs
Modified Immune Cells (AFM13-NK) and A Monoclonal Antibody (AFM13) in Treating Patients With Recurrent or Refractory CD30 Positive Hodgkin or Non-Hodgkin Lymphomas	NCT04074746	Phase I–II	AFM13-NK	Cord blood natural killer cells + AFM13
A Study of Pembrolizumab/Vibostolimab (MK-7684A) in Relapsed/Refractory Hematological Malignancies (MK-7684A-004, KEYVIBE-004)	NCT05005442	Phase II	Vibostolimab + Pembrolizumab	Anti-TIGIT + anti-PD1
Nivolumab With Ruxolitinib in Relapsed or Refractory Classical Hodgkin Lymphoma	NCT03681561	Phase I–II	Ruxolitinib + Nivolumab	JAK inhibitor + anti-PD1
A Study to Evaluate the Efficacy and Safety of a Sintilimab Plus ICE Regimen Versus ICE Regimen in Classic Hodgkin’s Lymphoma Patients (cHL) Who Have Failed First-line Standard Chemotherapy	NCT04044222	Phase III	Sintilimab + ICE vs. ICE	Anti-PD1 + Chemotherapy
Tislelizumab in Participants With Relapsed or Refractory Classical Hodgkin Lymphoma	NCT04318080	Phase II	Tislelizumab	Anti-PD1
Tislelizumab, Gemcitabine and Cisplatin for R/R Hodgkin Lymphoma Followed by Tislelizumab Consolidation in Patients in Metabolic Complete Remission	NCT05502250	Phase II	Tislelizumab + Gemcitabine + Cisplatin	Anti-PD1 + Chemotherapy
Tislelizumab Monotherapy Versus Salvage Chemotherapy for Relapsed/Refractory Classical Hodgkin Lymphoma	NCT04486391	Phase III	Tislelizumab vs. chemotherapy	Anti-PD1
PD-1 Inhibitor Combined With Decitabine Followed by ASCT as Second-line Therapy for Relapsed or Refractory Classic Hodgkin’s Lymphoma	NCT05137886	Phase II	Anti-PD1 + Azacitidine followed by ASCT	Anti-PD1 + hypomethylating agent
Study of Magrolimab and Pembrolizumab in Relapsed or Refractory Classic Hodgkin Lymphoma	NCT04788043	Phase II	Magrolimab + Pembrolizumab	Anti-CD47 + anti PD1
CC-486 and Nivolumab for the Treatment of Hodgkin Lymphoma Refractory to PD-1 Therapy or Relapsed	NCT05162976	Phase I	CC-486 (oral azacitidine) + Nivolumab	hypomethylating agent + anti-PD1
SHR1701 Alone or in Combination With SHR2554 in Relapsed or Refractory Classical Hodgkin Lymphoma	NCT05896046	Phase I–II	SHR1701 or SHR2554 + SHR1701	Anti-PDL1 with or without EZH2 inhibitor
Pembrolizumab and Vorinostat in Patients With Relapsed or Refractory DLBCL, FCL or HL.	NCT03150329	Phase I	Pembrolizumab + Vorinostat	HDACi + anti-PD1
Addition of Chidamide to the Combination Treatment of Decitabine Plus Camrelizumab in Combination Treatment Resistant/Relapsed Patients With Classical Hodgkin Lymphoma	NCT04233294	Phase II	Tucidinostat + Azacitidine + Camrelizumab	HDACi + hypometilating agent + anti-PD1
The Clinical Trial of Chidamide +Decitabine +Camrelizumab Versus Decitabine+Camrelizumab in Anti-PD-1 Antibody Resistant Patients With Classical Hodgkin Lymphoma.	NCT04514081	Phase II	Tucidinostat + Azacitidine + Camrelizumab vs. Azacitidine + Camrelizumab	HDACi + hypometilating agent + anti-PD1
A Phase II Study of the Combination of Azacitidine and Pembrolizumab for Patients Relapsed/Refractory Hodgkin’s Lymphoma	NCT05355051	Phase II	Azacitidine + Pembrolizumab	Hypometilating agent + anti-PD1
Azacitidine Plus PD-1 Therapy for R/R Hodgkin Lymphoma	NCT06190067	Phase II	Azacitidine + anti PD1	Hypometilating agent + anti-PD1
SHR-1210 Alone or in Combination With Decitabine in Relapsed or Refractory Hodgkin Lymphoma	NCT03250962	Phase II	SHR-1210 + Decitabine	Hypometilating agent + anti-PD1
Camrelizumab Plus Decitabine in Anti-PD-1 Treatment-nïive Patients With Relapsed/Refractory Classical Hodgkin Lymphoma	NCT04510610	Phase II–III	Camrelizumab + Decitabine	Hypometilating agent + anti-PD1
PD-1 Inhibitor Combined With Decitabine Followed by ASCT as Second-line Therapy for Relapsed or Refractory Classic Hodgkin’s Lymphoma	NCT05137886	Phase II	Anti PD1 + Decitabine + ASCT	Hypometilating agent + anti-PD1
Itacitinib + Everolimus in Hodgkin Lymphoma	NCT03697408	Phase I–II	Itacitinib + Everolimus	JAK1 inhibitor + mTOR inhibitor
CD30 CAR T Cells, Relapsed CD30 Expressing Lymphoma (RELY-30)	NCT02917083	Phase I	CAR T-cells	CD30 CAR T-cells
A Multicenter Clinical Study on the Safety and Effectiveness of CAR-T in the Treatment of Relapsed/Refractory Hodgkin’s Lymphoma	NCT04665063	Not applicable	CAR T-cells	CD30 CAR T-cells
Phase 2 Study Evaluating Autologous CD30.CAR-T Cells in Patients With Relapsed/Refractory Hodgkin Lymphoma (CHARIOT)	NCT04268706	Phase II	CAR T cells	CD30 CAR T-cells
Autologous CD30.CAR-T in Combination With Nivolumab in cHL Patients After Failure of Frontline Therapy	NCT05352828	Phase I	CAR T-cells + Nivolumab	CD30 CAR T-cells + anti-PD1
Allogeneic CD30.CAR-EBVSTs in Patients With Relapsed or Refractory CD30-Positive Lymphomas	NCT04288726	Phase I	CAR T-cells	Allogeneic CD30 EBV-specific CAR T-cells
Allogeneic CD30 Chimeric Antigen Receptor Epstein-Barr Virus-Specific T Lymphocytes in Relapsed or Refractory CD30-Positive Lymphomas	NCT04952584	Phase I	CAR T-cells	Allogeneic CD30 EBV-specific CAR T-cells
Study of PD-1 Inhibitors After CD30.CAR T Cell Therapy in Relapsed/Refractory Hodgkin Lymphoma	NCT04134325	Early Phase I	Anti-PD1	Anti-PD1 after CD30 CAR T-cells
Constitutive IL7R (C7R) Modified Banked Allogeneic CD30.CAR EBVSTs for CD30-Positive Lymphomas	NCT06176690	Phase I	CAR T-cells	Allogeneic CD30 EBV-specific CAR T-cells
CD30 Targeted CAR-T in Treating CD30-Expressing Lymphomas	NCT03383965	Phase I	CAR T-cells	CD30 CAR T-cells
CD30-directed Chimeric Antigen Receptor T (CART30) Therapy in Relapsed and Refractory CD30 Positive Lymphomas	NCT02259556	Phase I–II	CAR T-cells	CD30 CAR T-cells
Study of CD30 CAR for Relapsed/Refractory CD30+ HL and CD30+ NHL	NCT02690545	Phase I–II	CAR T-cells	CD30 CAR T-cells
Administration of T Lymphocytes for Prevention of Relapse of Lymphomas	NCT02663297	Phase I	CAR T-cells	CD30 CAR T-cells
ATLCAR.CD30.CCR4 for CD30+ HL ATLCAR.CD30.CCR4 Cells	NCT06090864	Phase I–II	CAR T-cells	CD30 and CCR4 CAR T-cells
Study of CAR-T Cells Expressing CD30 and CCR4 for r/r CD30+ HL and CTCL	NCT03602157	Phase I	CAR T-cells	CD30 and CCR4 CAR T-cells

ASCT (Autologous Stem Cell Transplant), CAR (Chimeric Antigen Receptor), EBV (Epstein Barr Virus), EBVSTs (EBV-specific T cells), HDACi (Hystone Deacetylases inhibitor), ICE (Ifosfamide, Carboplatin, Etoposide), NK (Natural Killer), PD1 (Programmed Death receptor 1), PDL1 (Programmed Death ligand 1).

## References

[1] Hodgkin Lymphoma—Cancer Stat Facts. https://seer.cancer.gov/statfacts/html/hodg.html.

[2] Engert A. (2016). ABVD or BEACOPP for Advanced Hodgkin Lymphoma. J. Clin. Oncol..

[3] Bonadonna G., Zucali R., Monfardini S., de Lena M., Uslenchi C. (1975). Combination chemotherapy of hodgkin’s disease with adriamycin, bleomycin, vinblastine, and imidazole carboxamide versus mopp. Cancer.

[4] Evens A.M., Hutchings M., Diehl V. (2008). Treatment of Hodgkin Lymphoma: The Past, Present, and Future. Nat. Clin. Pract. Oncol..

[5] Küppers R. (2012). New Insights in the Biology of Hodgkin Lymphoma. Hematol. Am. Soc. Hematol. Educ. Program..

[6] Mathas S., Hartmann S., Küppers R. (2016). Hodgkin Lymphoma: Pathology and Biology. Semin. Hematol..

[7] Fromm J.R., Thomas A., Wood B.L. (2009). Flow Cytometry Can Diagnose Classical Hodgkin Lymphoma in Lymph Nodes with High Sensitivity and Specificity. Am. J. Clin. Pathol..

[8] Skinnider B.F., Elia A.J., Gascoyne R.D., Patterson B., Trumper L., Kapp U., Mak T.W. (2002). Signal Transducer and Activator of Transcription 6 Is Frequently Activated in Hodgkin and Reed-Sternberg Cells of Hodgkin Lymphoma. Blood.

[9] Voltin C.A., Mettler J., van Heek L., Goergen H., Muller H., Baues C., Keller U., Meissner J., Trautmann-Grill K., Kerkhoff A. (2021). Early Response to First-Line Anti-PD-1 Treatment in Hodgkin Lymphoma: A PET-Based Analysis from the Prospective, Randomized Phase II NIVAHL Trial. Clin. Cancer Res..

[10] van der Weyden C.A., Pileri S.A., Feldman A.L., Whisstock J., Prince H.M. (2017). Understanding CD30 Biology and Therapeutic Targeting: A Historical Perspective Providing Insight into Future Directions. Blood Cancer J..

[11] Falini B., Pileri S., Pizzolo G., Dürkop H., Flenghi L., Stirpe F., Martelli M.F., Stein H. (1995). CD30 (Ki-1) Molecule: A New Cytokine Receptor of the Tumor Necrosis Factor Receptor Superfamily as a Tool for Diagnosis and Immunotherapy. Blood.

[12] Stein H., Mason D., Gerdes J., O’Connor N., Wainscoat J., Pallesen G., Gatter K., Falini B., Delsol G., Lemke H. (1985). The Expression of the Hodgkin’s Disease Associated Antigen Ki-1 in Reactive and Neoplastic Lymphoid Tissue: Evidence That Reed-Sternberg Cells and Histiocytic Malignancies Are Derived from Activated Lymphoid Cells. Blood.

[13] Zheng B., Flumara P., Li Y.V., Georgakis G., Snell V., Younes M., Vauthey J.N., Carbone A., Younes A. (2003). MEK/ERK Pathway Is Aberrantly Active in Hodgkin Disease: A Signaling Pathway Shared by CD30, CD40, and RANK That Regulates Cell Proliferation and Survival. Blood.

[14] Mir S.S., Richter B.W.M., Duckett C.S. (2000). Differential Effects of CD30 Activation in Anaplastic Large Cell Lymphoma and Hodgkin Disease Cells. Blood.

[15] Schirrmann T., Steinwand M., Wezler X., Ten Haaf A., Tur M.K., Barth S. (2014). CD30 as a Therapeutic Target for Lymphoma. BioDrugs.

[16] Gerber H.P. (2010). Emerging Immunotherapies Targeting CD30 in Hodgkin’s Lymphoma. Biochem. Pharmacol..

[17] Aldinucci D., Gloghini A., Pinto A., De Filippi R., Carbone A. (2010). The Classical Hodgkin’s Lymphoma Microenvironment and Its Role in Promoting Tumour Growth and Immune Escape. J. Pathol..

[18] Rigo A., Vinante F. (2017). Flow Cytometry Analysis of Receptor Internalization/Shedding. Cytom. B Clin. Cytom..

[19] Younes A., Bartlett N.L., Leonard J.P., Kennedy D.A., Lynch C.M., Sievers E.L., Forero-Torres A. (2010). Brentuximab Vedotin (SGN-35) for Relapsed CD30-Positive Lymphomas. N. Engl. J. Med..

[20] Younes A., Gopal A.K., Smith S.E., Ansell S.M., Rosenblatt J.D., Savage K.J., Ramchandren R., Bartlett N.L., Cheson B.D., De Vos S. (2012). Results of a Pivotal Phase II Study of Brentuximab Vedotin for Patients with Relapsed or Refractory Hodgkin’s Lymphoma. J. Clin. Oncol..

[21] Adcetris European Medicines Agency. https://www.ema.europa.eu/en/medicines/human/EPAR/adcetris.

[22] Drug Approval Package: ADCETRIS (Brentuximab Vedotin) NDA #125399. https://www.accessdata.fda.gov/drugsatfda_docs/nda/2011/125399_adcetris_toc.cfm.

[23] Cheson B.D., Fisher R.I., Barrington S.F., Cavalli F., Schwartz L.H., Zucca E., Lister T.A. (2014). Recommendations for Initial Evaluation, Staging, and Response Assessment of Hodgkin and Non-Hodgkin Lymphoma: The Lugano Classification. J. Clin. Oncol..

[24] Onishi M., Graf S.A., Holmberg L., Behnia S., Shustov A.R., Schiavo K., Philip M., Libby E.N., Cassaday R.D., Pagel J.M. (2015). Brentuximab Vedotin Administered to Platinum-Refractory, Transplant-Naïve Hodgkin Lymphoma Patients Can Increase the Proportion Achieving FDG PET Negative Status. Hematol. Oncol..

[25] Zinzani P.L., Pellegrini C., Cantonetti M., Re A., Pinto A., Pavone V., Rigacci L., Celli M., Broccoli A., Argnani L. (2015). Brentuximab Vedotin in Transplant-Naïve Relapsed/Refractory Hodgkin Lymphoma: Experience in 30 Patients. Oncologist.

[26] Moskowitz C.H., Walewski J., Nademanee A., Masszi T., Agura E., Holowiecki J., Abidi M.H., Chen A.I., Stiff P., Viviani S. (2018). Five-Year PFS from the AETHERA Trial of Brentuximab Vedotin for Hodgkin Lymphoma at High Risk of Progression or Relapse. Blood.

[27] Chen R., Palmer J.M., Thomas S.H., Tsai N.C., Farol L., Nademanee A., Forman S.J., Gopal A.K. (2012). Brentuximab Vedotin Enables Successful Reduced-Intensity Allogeneic Hematopoietic Cell Transplantation in Patients with Relapsed or Refractory Hodgkin Lymphoma. Blood.

[28] Carlo-Stella C., Ricci F., Dalto S., Mazza R., Malagola M., Patriarca F., Viviani S., Russo D., Giordano L., Castagna L. (2015). Brentuximab Vedotin in Patients with Hodgkin Lymphoma and a Failed Allogeneic Stem Cell Transplantation: Results From a Named Patient Program at Four Italian Centers. Oncologist.

[29] LaCasce A.S., Gregory Bociek R., Sawas A., Caimi P., Agura E., Matous J., Ansell S.M., Crosswell H.E., Islas-Ohlmayer M., Behler C. (2018). Brentuximab Vedotin plus Bendamustine: A Highly Active First Salvage Regimen for Relapsed or Refractory Hodgkin Lymphoma. Blood.

[30] O’Connor O.A., Lue J.K., Sawas A., Amengual J.E., Deng C., Kalac M., Falchi L., Marchi E., Turenne I., Lichtenstein R. (2018). Brentuximab Vedotin plus Bendamustine in Relapsed or Refractory Hodgkin’s Lymphoma: An International, Multicentre, Single-Arm, Phase 1–2 Trial. Lancet Oncol..

[31] Broccoli A., Argnani L., Botto B., Corradini P., Pinto A., Re A., Vitolo U., Fanti S., Stefoni V., Zinzani P.L. (2019). First Salvage Treatment with Bendamustine and Brentuximab Vedotin in Hodgkin Lymphoma: A Phase 2 Study of the Fondazione Italiana Linfomi. Blood Cancer J..

[32] Greenwald R.J., Freeman G.J., Sharpe A.H. (2005). The B7 Family Revisited. Annu. Rev. Immunol..

[33] Zou W., Chen L. (2008). Inhibitory B7-Family Molecules in the Tumour Microenvironment. Nat. Rev. Immunol..

[34] Zak K.M., Grudnik P., Magiera K., Dömling A., Dubin G., Holak T.A. (2017). Structural Biology of the Immune Checkpoint Receptor PD-1 and Its Ligands PD-L1/PD-L2. Structure.

[35] Alsaab H.O., Sau S., Alzhrani R., Tatiparti K., Bhise K., Kashaw S.K., Iyer A.K. (2017). PD-1 and PD-L1 Checkpoint Signaling Inhibition for Cancer Immunotherapy: Mechanism, Combinations, and Clinical Outcome. Front. Pharmacol..

[36] Dong H., Zhu G., Tamada K., Chen L. (1999). B7-H1, a Third Member of the B7 Family, Co-Stimulates T-Cell Proliferation and Interleukin-10 Secretion. Nat. Med..

[37] Keir M.E., Butte M.J., Freeman G.J., Sharpe A.H. (2008). PD-1 and Its Ligands in Tolerance and Immunity. Annu. Rev. Immunol..

[38] Chemnitz J.M., Parry R.V., Nichols K.E., June C.H., Riley J.L. (2004). SHP-1 and SHP-2 Associate with Immunoreceptor Tyrosine-Based Switch Motif of Programmed Death 1 upon Primary Human T Cell Stimulation, but Only Receptor Ligation Prevents T Cell Activation. J. Immunol..

[39] Yokosuka T., Takamatsu M., Kobayashi-Imanishi W., Hashimoto-Tane A., Azuma M., Saito T. (2012). Programmed Cell Death 1 Forms Negative Costimulatory Microclusters That Directly Inhibit T Cell Receptor Signaling by Recruiting Phosphatase SHP2. J. Exp. Med..

[40] Carey C.D., Gusenleitner D., Lipschitz M., Roemer M.G.M., Stack E.C., Gjini E., Hu X., Redd R., Freeman G.J., Neuberg D. (2017). Topological Analysis Reveals a PD-L1-Associated Microenvironmental Niche for Reed-Sternberg Cells in Hodgkin Lymphoma. Blood.

[41] Opdivo European Medicines Agency. https://www.ema.europa.eu/en/medicines/human/EPAR/opdivo.

[42] Nivolumab (Opdivo) for Hodgkin Lymphoma FDA. https://www.fda.gov/drugs/resources-information-approved-drugs/nivolumab-opdivo-hodgkin-lymphoma.

[43] Ansell S.M., Lesokhin A.M., Borrello I., Halwani A., Scott E.C., Gutierrez M., Schuster S.J., Millenson M.M., Cattry D., Freeman G.J. (2015). PD-1 Blockade with Nivolumab in Relapsed or Refractory Hodgkin’s Lymphoma. N. Engl. J. Med..

[44] Younes A., Santoro A., Shipp M., Zinzani P.L., Timmerman J.M., Ansell S., Armand P., Fanale M., Ratanatharathorn V., Kuruvilla J. (2016). Nivolumab for Classical Hodgkin’s Lymphoma after Failure of Both Autologous Stem-Cell Transplantation and Brentuximab Vedotin: A Multicentre, Multicohort, Single-Arm Phase 2 Trial. Lancet Oncol..

[45] Ansell S.M., Bröckelmann P.J., von Keudell G., Lee H.J., Santoro A., Zinzani P.L., Collins G.P., Cohen J.B., de Boer J.P., Kuruvilla J. (2023). Nivolumab for Relapsed/Refractory Classical Hodgkin Lymphoma: 5-Year Survival from the Pivotal Phase 2 CheckMate 205 Study. Blood Adv..

[46] Harker-Murray P., Mauz-Körholz C., Leblanc T., Mascarin M., Michel G., Cooper S., Beishuizen A., Leger K.J., Amoroso L., Buffardi S. (2023). Nivolumab and Brentuximab Vedotin with or without Bendamustine for R/R Hodgkin Lymphoma in Children, Adolescents, and Young Adults. Blood.

[47] Keytruda European Medicines Agency. https://www.ema.europa.eu/en/medicines/human/EPAR/keytruda.

[48] FDA Extends Approval of Pembrolizumab for Classical Hodgkin Lymphoma FDA. https://www.fda.gov/drugs/resources-information-approved-drugs/fda-extends-approval-pembrolizumab-classical-hodgkin-lymphoma.

[49] Armand P., Shipp M.A., Ribrag V., Michot J.M., Zinzani P.L., Kuruvilla J., Snyder E.S., Ricart A.D., Balakumaran A., Rose S. (2016). Programmed Death-1 Blockade with Pembrolizumab in Patients with Classical Hodgkin Lymphoma after Brentuximab Vedotin Failure. J. Clin. Oncol..

[50] Chen R., Zinzani P.L., Fanale M.A., Armand P., Johnson N.A., Brice P., Radford J., Ribrag V., Molin D., Vassilakopoulos T.P. (2017). Phase II Study of the Efficacy and Safety of Pembrolizumab for Relapsed/Refractory Classic Hodgkin Lymphoma. J. Clin. Oncol..

[51] Chen R., Zinzani P.L., Lee H.J., Armand P., Johnson N.A., Brice P., Radford J., Ribrag V., Molin D., Vassilakopoulos T.P. (2019). Pembrolizumab in Relapsed or Refractory Hodgkin Lymphoma: 2-Year Follow-up of KEYNOTE-087. Blood.

[52] Herbaux C., Gauthier J., Brice P., Drumez E., Ysebaert L., Doyen H., Fornecker L., Bouabdallah K., Manson G., Ghesquières H. (2017). Efficacy and Tolerability of Nivolumab after Allogeneic Transplantation for Relapsed Hodgkin Lymphoma. Blood.

[53] Shi Y., Su H., Song Y., Jiang W., Sun X., Qian W., Zhang W., Gao Y., Jin Z., Zhou J. (2019). Safety and Activity of Sintilimab in Patients with Relapsed or Refractory Classical Hodgkin Lymphoma (ORIENT-1): A Multicentre, Single-Arm, Phase 2 Trial. Lancet Haematol..

[54] Song Y., Gao Q., Zhang H., Fan L., Zhou J., Zou D., Li W., Yang H., Liu T., Wang Q. (2020). Treatment of Relapsed or Refractory Classical Hodgkin Lymphoma with the Anti-PD-1, Tislelizumab: Results of a Phase 2, Single-Arm, Multicenter Study. Leukemia.

[55] Lin N., Zhang M., Bai H., Liu H., Cui J., Ke X., Zhang H., Liu L., Yan D., Jiang Y. (2022). Efficacy and Safety of GLS-010 (Zimberelimab) in Patients with Relapsed or Refractory Classical Hodgkin Lymphoma: A Multicenter, Single-Arm, Phase II Study. Eur. J. Cancer.

[56] Herrera A.F., Burton C., Radford J., Miall F., Townsend W., Santoro A., Zinzani P.L., Lewis D., Fowst C., Brar S. (2021). Avelumab in Relapsed/Refractory Classical Hodgkin Lymphoma: Phase 1b Results from the JAVELIN Hodgkins Trial. Blood Adv..

[57] Rossi C., Gilhodes J., Maerevoet M., Herbaux C., Morschhauser F., Brice P., Garciaz S., Borel C., Ysebaert L., Obéric L. (2018). Efficacy of Chemotherapy or Chemo-Anti-PD-1 Combination after Failed Anti-PD-1 Therapy for Relapsed and Refractory Hodgkin Lymphoma: A Series from Lysa Centers. Am. J. Hematol..

[58] Carreau N.A., Diefenbach C.S. (2019). Immune Targeting of the Microenvironment in Classical Hodgkin’s Lymphoma: Insights for the Hematologist. Ther. Adv. Hematol..

[59] Casadei B., Argnani L., Morigi A., Lolli G., Broccoli A., Pellegrini C., Nanni L., Stefoni V., Coppola P.E., Carella M. (2020). Effectiveness of Chemotherapy after Anti-PD-1 Blockade Failure for Relapsed and Refractory Hodgkin Lymphoma. Cancer Med..

[60] ADCETRIS® (Brentuximab Vedotin)—Seagen. https://www.seagen.com/medicines/adcetris.

[61] Straus D.J., Długosz-Danecka M., Connors J.M., Alekseev S., Illés Á., Picardi M., Lech-Maranda E., Feldman T., Smolewski P., Savage K.J. (2021). Brentuximab Vedotin with Chemotherapy for Stage III or IV Classical Hodgkin Lymphoma (ECHELON-1): 5-Year Update of an International, Open-Label, Randomised, Phase 3 Trial. Lancet Haematol..

[62] Ramchandren R., Advani R.H., Ansell S.M., Bartlett N.L., Chen R., Connors J.M., Feldman T., Forero-Torres A., Friedberg J.W., Gopal A.K. (2019). Brentuximab Vedotin plus Chemotherapy in North American Subjects with Newly Diagnosed Stage III or IV Hodgkin Lymphoma. Clin. Cancer Res..

[63] Suri A., Mould D.R., Song G., Collins G.P., Endres C.J., Gomez-Navarro J., Venkatakrishnan K. (2019). Population Pharmacokinetic Modeling and Exposure-Response Assessment for the Antibody-Drug Conjugate Brentuximab Vedotin in Hodgkin’s Lymphoma in the Phase III ECHELON-1 Study. Clin. Pharmacol. Ther..

[64] Cai Q., Xia Y., Liu P., Zhang Y., Zou Q., Cai J. (2022). Real-World Study Evaluating the Efficacy of Frontline Brentuximab Vedotin Plus Chemotherapy in Newly Diagnosed Patients with Hodgkin Lymphoma: A Retrospective Analysis. Blood.

[65] Ansell S.M., Radford J., Connors J.M., Długosz-Danecka M., Kim W.-S., Gallamini A., Ramchandren R., Friedberg J.W., Advani R., Hutchings M. (2022). Overall Survival with Brentuximab Vedotin in Stage III or IV Hodgkin’s Lymphoma. N. Engl. J. Med..

[66] Fornecker L.M., Lazarovici J., Aurer I., Casasnovas R.O., Gac A.C., Bonnet C., Bouabdallah K., Feugier P., Specht L., Molina L. (2023). Brentuximab Vedotin Plus AVD for First-Line Treatment of Early-Stage Unfavorable Hodgkin Lymphoma (BREACH): A Multicenter, Open-Label, Randomized, Phase II Trial. J. Clin. Oncol..

[67] Abramson J.S., Bengston E., Redd R., Barnes J.A., Takvorian T., Sokol L., Lansigan F., Armand P., Shah B., Jacobsen E. (2023). Brentuximab Vedotin plus Doxorubicin and Dacarbazine in Nonbulky Limited-Stage Classical Hodgkin Lymphoma. Blood Adv..

[68] Kumar A., Casulo C., Advani R.H., Budde E., Barr P.M., Batlevi C.L., Caron P., Constine L.S., Dandapani S.V., Drill E. (2021). Brentuximab Vedotin Combined with Chemotherapy in Patients with Newly Diagnosed Early-Stage, Unfavorable-Risk Hodgkin Lymphoma. J. Clin. Oncol..

[69] Rubinstein P.G., Moore P.C., Rudek M.A., Henry D.H., Ramos J.C., Ratner L., Reid E., Sharon E., Noy A. (2018). Brentuximab Vedotin with AVD Shows Safety, in the Absence of Strong CYP3A4 Inhibitors, in Newly Diagnosed HIV-Associated Hodgkin Lymphoma. Aids.

[70] Rubinstein P.G., Moore P.C., Bimali M., Lee J.Y., Rudek M.A., Chadburn A., Ratner L., Henry D.H., Cesarman E., DeMarco C.E. (2023). Brentuximab Vedotin with AVD for Stage II-IV HIV-Related Hodgkin Lymphoma (AMC 085): Phase 2 Results from an Open-Label, Single Arm, Multicentre Phase 1/2 Trial. Lancet Haematol..

[71] Gibb A., Pirrie S.J., Linton K., Warbey V., Paterson K., Davies A.J., Collins G.P., Menne T., McKay P., Fields P.A. (2021). Results of a UK National Cancer Research Institute Phase II Study of Brentuximab Vedotin Using a Response-Adapted Design in the First-Line Treatment of Patients with Classical Hodgkin Lymphoma Unsuitable for Chemotherapy Due to Age, Frailty or Comorbidity (BREVITY). Br. J. Haematol..

[72] Forero-Torres A., Holkova B., Goldschmidt J., Chen R., Olsen G., Boccia R.V., Bordoni R.E., Friedberg J.W., Sharman J.P., Palanca-Wessels M.C. (2015). Phase 2 Study of Frontline Brentuximab Vedotin Monotherapy in Hodgkin Lymphoma Patients Aged 60 Years and Older. Blood.

[73] Yasenchak C.A., Bordoni R., Patel-Donnelly D., Larson T., Goldschmidt J., Boccia R.V., Cline V.J.M., Mamidipalli A., Liu J., Beck J.T. (2022). Brentuximab Vedotin in Frontline Therapy of Hodgkin Lymphoma in Patients with Significant Comorbidities Ineligible for Standard Chemotherapy (SGN35-015 Part E). Blood.

[74] Evens A.M., Advani R.H., Helenowski I.B., Jovanovic B.D., Winter J.N., Gordon L.I., Winte J.N., Gordon L.I., Smith S.M., Fanale M. (2018). Multicenter Phase II Study of Sequential Brentuximab Vedotin and Doxorubicin, Vinblastine, and Dacarbazine Chemotherapy for Older Patients With Untreated Classical Hodgkin Lymphoma. J. Clin. Oncol..

[75] Friedberg J.W., Forero-Torres A., Bordoni R.E., Cline V.J.M., Donnelly D.P., Flynn P.J., Olsen G., Chen R., Fong A., Wang Y. (2017). Frontline Brentuximab Vedotin in Combination with Dacarbazine or Bendamustine in Patients Aged ≥ 60 Years with HL. Blood.

[76] Evens A.M., Connors J.M., Younes A., Ansell S.M., Kim W.S., Radford J., Feldman T., Tuscano J., Savage K.J., Oki Y. (2022). Older Patients (Aged ≥ 60 Years) with Previously Untreated Advanced-Stage Classical Hodgkin Lymphoma: A Detailed Analysis from the Phase III ECHELON-1 Study. Haematologica.

[77] Eichenauer D.A., Plütschow A., Kreissl S., Sökler M., Hellmuth J.C., Meissner J., Mathas S., Topp M.S., Behringer K., Klapper W. (2017). Incorporation of Brentuximab Vedotin into First-Line Treatment of Advanced Classical Hodgkin’s Lymphoma: Final Analysis of a Phase 2 Randomised Trial by the German Hodgkin Study Group. Lancet Oncol..

[78] Borchmann P., Moccia A.A., Greil R., Schneider G., Hertzberg M., Schaub V., Hüttmann A., Keil F., Dierlamm J., Hänel M. (2023). Brecadd is non-inferior to ebeacopp in patients with advanced stage classical hodgkin lymphoma: Efficacy results of the GHSG Phase III HD21 trial. Hematol. Oncol..

[79] Bröckelmann P.J., Goergen H., Keller U., Meissner J., Ordemann R., Halbsguth T.V., Sasse S., Sökler M., Kerkhoff A., Mathas S. (2020). Efficacy of Nivolumab and AVD in Early-Stage Unfavorable Classic Hodgkin Lymphoma: The Randomized Phase 2 German Hodgkin Study Group NIVAHL Trial. JAMA Oncol..

[80] Bröckelmann P.J., Bühnen I., Meissner J., Trautmann-Grill K., Herhaus P., Halbsguth T.V., Schaub V., Kerkhoff A., Mathas S., Bormann M. (2023). Nivolumab and Doxorubicin, Vinblastine, and Dacarbazine in Early-Stage Unfavorable Hodgkin Lymphoma: Final Analysis of the Randomized German Hodgkin Study Group Phase II NIVAHL Trial. J. Clin. Oncol..

[81] Ramchandren R., Domingo-Domènech E., Rueda A., Trněný M., Feldman T.A., Lee H.J., Provencio M., Sillaber C., Cohen J.B., Savage K.J. (2019). Nivolumab for Newly Diagnosed Advanced-Stage Classic Hodgkin Lymphoma: Safety and Efficacy in the Phase II CheckMate 205 Study. J. Clin. Oncol..

[82] Rutherford S.C., Li H., Herrera A.F., Leblanc M., Ahmed S., Davison K.L., Casulo C., Bartlett N.L., Tuscano J.M., Hess B. (2023). Nivolumab-AVD Is Better Tolerated and Improves Progression-Free Survival Compared to Bv-AVD in Older Patients (Aged ≥ 60 Years) with Advanced Stage Hodgkin Lymphoma Enrolled on SWOG S1826. Blood.

[83] Allen P.B., Savas H., Evens A.M., Advani R.H., Palmer B., Pro B., Karmali R., Mou E., Bearden J., Dillehay G. (2021). Pembrolizumab Followed by AVD in Untreated Early Unfavorable and Advanced-Stage Classical Hodgkin Lymphoma. Blood.

[84] Allen P.B., Lu X., Chen Q., O’Shea K., Chmiel J.S., Slonim L.B., Sukhanova M., Savas H., Evens A.M., Advani R. (2023). Sequential Pembrolizumab and AVD Are Highly Effective at Any PD-L1 Expression Level in Untreated Hodgkin Lymphoma. Blood Adv..

[85] Lynch R.C., Ujjani C.S., Poh C., Warren E.H., Smith S.D., Shadman M., Till B., Raghunathan V.M., Alig S., Alizadeh A.A. (2023). Concurrent Pembrolizumab with AVD for Untreated Classic Hodgkin Lymphoma. Blood.

[86] Cheson B.D., Bartlett N.L., LaPlant B., Lee H.J., Advani R.J., Christian B., Diefenbach C.S., Feldman T.A., Ansell S.M. (2020). Brentuximab Vedotin plus Nivolumab as First-Line Therapy in Older or Chemotherapy-Ineligible Patients with Hodgkin Lymphoma (ACCRU): A Multicentre, Single-Arm, Phase 2 Trial. Lancet Haematol..

[87] Friedberg J.W., Bordoni R., Patel-Donnelly D., Larson T., Goldschmidt J., Boccia R., Cline V.J.M., Mamidipalli A., Liu J., Akyol A. (2024). Brentuximab Vedotin with Dacarbazine or Nivolumab as Frontline CHL Therapy for Older Patients Ineligible for Chemotherapy. Blood.

[88] Park S.I., Ansell S.M., Giri S., Svoboda J., Smith S.D., Feldman T., Budde E.L., Ness A.J., Choi Y., Bierman P.J. (2022). Frontline PET-Directed Therapy with Brentuximab Vedotin Plus AVD Followed By Nivolumab Consolidation in Patients with Limited Stage Hodgkin Lymphoma. Blood.

[89] Abramson J.S., Straus D.J., Bartlett N.L., Burke J.M., Lynch R.C., Domingo Domenech E., Hess B., Schuster S.R., Linhares Y., Ramchandren R. (2023). Brentuximab Vedotin, Nivolumab, Doxorubicin, and Dacarbazine (AN+AD) for Early-Stage Classical Hodgkin Lymphoma (SGN35-027 Part C). Blood.

[90] Lee H.J., Flinn I.W., Melear J., Ramchandren R., Friedman J., Burke J.M., Linhares Y., Gonzales P.A., Raval M., Chintapatla R. (2023). Brentuximab Vedotin, Nivolumab, Doxorubicin, and Dacarbazine for Advanced Stage Classical Hodgkin Lymphoma: Efficacy and Safety Results from the Single Arm Phase 2 Study. Blood.

[91] Flynn M.J., Hartley J.A. (2017). The Emerging Role of Anti-CD25 Directed Therapies as Both Immune Modulators and Targeted Agents in Cancer. Br. J. Haematol..

[92] Facciabene A., Motz G.T., Coukos G. (2012). T Regulatory Cells: Key Players in Tumor Immune Escape and Angiogenesis. Cancer Res..

[93] Zammarchi F., Havenith K., Bertelli F., Vijayakrishnan B., Chivers S., van Berkel P.H. (2020). CD25-Targeted Antibody-Drug Conjugate Depletes Regulatory T Cells and Eliminates Established Syngeneic Tumors via Antitumor Immunity. J. Immunother. Cancer.

[94] Dyczkowski J., Herrera A.F., Carlo-Stella C., Zinzani P.L., Toukam M., Cruz H.G., Havenith K., Boni J., Wuerthner J., Pantano S. (2022). CD25, Soluble CD25, and CCL17 As Potential Predictors of Clinical Response to Camidanlumab Tesirine in Patients with Relapsed/Refractory Classical Hodgkin Lymphoma. Blood.

[95] Hamadani M., Collins G.P., Caimi P.F., Samaniego F., Spira A., Davies A., Radford J., Menne T., Karnad A., Zain J.M. (2021). Camidanlumab Tesirine in Relapsed/Refractory Lymphoma: A Phase 1, Multicenter, Open-Label, Dose-Escalation, Dose-Expansion Study. Lancet Haematol..

[96] Herrera A.F., Ansell S.M., Zinzani P.L., Radford J., Maddocks K.J., Pinto A., Collins G.P., Bachanova V., Bartlett N.L., Bence-Bruckler I. (2022). Exploratory Analysis of Factors Influencing Efficacy and Safety of Camidanlumab Tesirine: Data from the Open-Label, Multicenter, Phase 2 Study of Patients with Relapsed or Refractory Classical Hodgkin Lymphoma (R/R CHL). Blood.

[97] Carlo-Stella C., Ansell S., Zinzani P.L., Radford J., Maddocks K., Pinto A., Collins G.P., Bachanova V., Bartlett N., Bence-Bruckler I. (2022). S201: Camidanlumab tesirine: Updated efficacy and safety in an open-label, multicenter, phase 2 study of patients with relapsed or refractory classical hodgkin lymphoma (R/R CHL). Hemasphere.

[98] Himed S., Chung C., Dulmage B., Jaglowski S., Bond D., Maddocks K., Kaffenberger B.H. (2023). Development of Recalcitrant Cutaneous Eruptions in Patients with Relapsed/Refractory Hodgkin’s Lymphoma Undergoing Treatment with Camidanlumab Tesirine. J. Eur. Acad. Dermatol. Venereol..

[99] Jabeen A., Huang S., Hartley J.A., Van Berkel P.H., Zammarchi F. (2020). Combination of Camidanlumab Tesirine, a CD25-Targeted ADC, with Gemcitabine Elicits Synergistic Anti-Tumor Activity in Preclinical Tumor Models. Blood.

[100] Rousseau A., Parisi C., Barlesi F. (2023). Anti-TIGIT Therapies for Solid Tumors: A Systematic Review. ESMO Open.

[101] Rui S., Kong X., Liu J., Wang L., Wang X., Zou X., Zheng X., Ye F., Xu H., Li Z. (2022). The Landscape of TIGIT Target and Clinical Application in Diseases. MedComm Oncol..

[102] Yusuf R., Jemielita T., Marinello P. (2021). Safety and Efficacy of Vibostolimab and Pembrolizumab in Patients with Relapsed or Refractory Hematologic Malignancies: A Multicohort, Open-Label, Phase 2 Study. Blood.

[103] Aggarwal V., Workman C.J., Vignali D.A.A. (2023). LAG-3 as the Third Checkpoint Inhibitor. Nat. Immunol..

[104] Li Y., Ju M., Miao Y., Zhao L., Xing L., Wei M. (2023). Advancement of Anti-LAG-3 in Cancer Therapy. FASEB J..

[105] Timmerman J., Lavie D., Johnson N.A., Avigdor A., Borchmann P., Andreadis C., Bazargan A., Gregory G.P., Keane C., Tzoran I. (2023). Favezelimab in Combination with Pembrolizumab in Patients with Heavily Pretreated Anti-PD-1-Refractory Classical Hodgkin Lymphoma: Updated Analysis of an Open-Label Phase 1/2 Study. Blood.

[106] Johnson N.A., Lavie D., Borchmann P., Gregory G.P., Herrera A.F., Minuk L., Vucinic V., Armand P., Avigdor A., Gasiorowski R. (2023). Favezelimab in Combination with Pembrolizumab in Patients with Anti-PD-1-Naive Relapsed or Refractory Classical Hodgkin Lymphoma: Updated Analysis of an Open-Label Phase 1/2 Study. Blood.

[107] Lavie D., Timmerman J., García-Sanz R., Kim W.S., Kim T.M., Avigdor A., Dierickx D., Jagadeesh D., Molin D.L., Ozcan M. (2023). Open-Label, Randomized, Phase 3 Study of Coformulated Favezelimab and Pembrolizumab Versus Chemotherapy in Patients with Relapsed or Refractory Classical Hodgkin Lymphoma Refractory to Anti-PD-1 Therapy: Keyform-008. Blood.

[108] Jaiswal S., Jamieson C.H.M., Pang W.W., Park C.Y., Chao M.P., Majeti R., Traver D., van Rooijen N., Weissman I.L. (2009). CD47 Is Upregulated on Circulating Hematopoietic Stem Cells and Leukemia Cells to Avoid Phagocytosis. Cell.

[109] Chao M.P., Alizadeh A.A., Tang C., Myklebust J.H., Varghese B., Gill S., Jan M., Cha A.C., Chan C.K., Tan B.T. (2010). Anti-CD47 Antibody Synergizes with Rituximab to Promote Phagocytosis and Eradicate Non-Hodgkin Lymphoma. Cell.

[110] López-Pereira B., Fernández-Velasco A.A., Fernández-Vega I., Corte-Torres D., Quirós C., Villegas J.A., Palomo P., González S., González A.P., Payer Á. (2020). Expression of CD47 Antigen in Reed-Sternberg Cells as a New Potential Biomarker for Classical Hodgkin Lymphoma. Clin. Transl. Oncol..

[111] Steidl C., Lee T., Shah S.P., Farinha P., Han G., Nayar T., Delaney A., Jones S.J., Iqbal J., Weisenburger D.D. (2010). Tumor-Associated Macrophages and Survival in Classic Hodgkin’s Lymphoma. N. Engl. J. Med..

[112] Tan K.L., Scott D.W., Hong F., Kahl B.S., Fisher R.I., Bartlett N.L., Advani R.H., Buckstein R., Rimsza L.M., Connors J.M. (2012). Tumor-Associated Macrophages Predict Inferior Outcomes in Classic Hodgkin Lymphoma: A Correlative Study from the E2496 Intergroup Trial. Blood.

[113] Daver N.G., Vyas P., Kambhampati S., Al Malki M.M., Larson R.A., Asch A.S., Mannis G., Chai-Ho W., Tanaka T.N., Bradley T.J. (2023). Tolerability and Efficacy of the Anticluster of Differentiation 47 Antibody Magrolimab Combined with Azacitidine in Patients with Previously Untreated AML: Phase Ib Results. J. Clin. Oncol..

[114] Sallman D.A., Al Malki M.M., Asch A.S., Wang E.S., Jurcic J.G., Bradley T.J., Flinn I.W., Pollyea D.A., Kambhampati S., Tanaka T.N. (2023). Magrolimab in Combination with Azacitidine in Patients with Higher-Risk Myelodysplastic Syndromes: Final Results of a Phase Ib Study. J. Clin. Oncol..

[115] Advani R., Flinn I., Popplewell L., Forero A., Bartlett N.L., Ghosh N., Kline J., Roschewski M., LaCasce A., Collins G.P. (2018). CD47 Blockade by Hu5F9-G4 and Rituximab in Non-Hodgkin’s Lymphoma. N. Engl. J. Med..

[116] Kerbauy L.N., Marin N.D., Kaplan M., Banerjee P.P., Berrien-Elliott M.M., Becker-Hapak M., Basar R., Foster M., Melo L.G., Neal C.C. (2021). Combining AFM13, a Bispecific CD30/CD16 Antibody, with Cytokine-Activated Blood and Cord Blood-Derived NK Cells Facilitates CAR-like Responses Against CD30+ Malignancies. Clin. Cancer Res..

[117] Pahl J.H.W., Koch J., Gotz J.J., Arnold A., Reusch U., Gantke T., Rajkovic E., Treder M., Cerwenka A. (2018). CD16A Activation of NK Cells Promotes NK Cell Proliferation and Memory-Like Cytotoxicity against Cancer Cells. Cancer Immunol. Res..

[118] Moskowitz A., Harstrick A., Emig M., Overesch A., Pinto S., Ravenstijn P., Schlüter T., Rubel J., Rebscher H., Graefe T. (2023). AFM13 in Combination with Allogeneic Natural Killer Cells (AB-101) in Relapsed or Refractory Hodgkin Lymphoma and CD30 + Peripheral T-Cell Lymphoma: A Phase 2 Study (LuminICE). Blood.

[119] Rothe A., Sasse S., Topp M.S., Eichenauer D.A., Hummel H., Reiners K.S., Dietlein M., Kuhnert G., Kessler J., Buerkle C. (2015). A Phase 1 Study of the Bispecific Anti-CD30/CD16A Antibody Construct AFM13 in Patients with Relapsed or Refractory Hodgkin Lymphoma. Blood.

[120] Sasse S., Bröckelmann P.J., Momotow J., Plütschow A., Hüttmann A., Basara N., Koenecke C., Martin S., Bentz M., Grosse-Thie C. (2022). AFM13 in Patients with Relapsed or Refractory Classical Hodgkin Lymphoma: Final Results of an Open-Label, Randomized, Multicenter Phase II Trial. Leuk. Lymphoma.

[121] Bartlett N.L., Herrera A.F., Domingo-Domenech E., Mehta A., Forero-Torres A., Garcia-Sanz R., Armand P., Devata S., Izquierdo A.R., Lossos I.S. (2020). A Phase 1b Study of AFM13 in Combination with Pembrolizumab in Patients with Relapsed or Refractory Hodgkin Lymphoma. Blood.

[122] Chen R., Hou J., Newman E., Kim Y., Donohue C., Liu X., Thomas S.H., Forman S.J., Kane S.E. (2015). CD30 Downregulation, MMAE Resistance, and MDR1 Upregulation Are All Associated with Resistance to Brentuximab Vedotin. Mol. Cancer Ther..

[123] Reusch U., Ellwanger K., Fucek I., Müller T., Schniegler-Mattox U., Pahl J., Tesar M., Koch J. (2021). Cryopreserved CAR-like NK Cells Pre-Complexed with the CD30/CD16A Bispecific Innate Cell Engager (ICE®) AFM13 for the Treatment of CD30 + Malignancies. Blood.

[124] Nieto Y., Banerjee P., Kaur I., Griffin L., Barnett M., Ganesh C., Borneo Z., Bassett R.L., Kerbauy L.N., Basar R. (2023). Innate Cell Engager (ICE®) AFM13 Combined with Preactivated and Expanded (P+E) Cord Blood (CB)-Derived Natural Killer (NK) Cells for Patients with Refractory CD30-Positive Lymphomas: Final Results. Blood.

[125] Costa P.M.d.S., Sales S.L.A., Pinheiro D.P., Pontes L.Q., Maranhão S.S., Pessoa C.d.Ó., Furtado G.P., Furtado C.L.M. (2023). Epigenetic Reprogramming in Cancer: From Diagnosis to Treatment. Front. Cell Dev. Biol..

[126] Ammerpohl O., Haake A., Pellissery S., Giefing M., Richter J., Balint B., Kulis M., Le J., Bibikova M., Drexler H.G. (2012). Array-Based DNA Methylation Analysis in Classical Hodgkin Lymphoma Reveals New Insights into the Mechanisms Underlying Silencing of B Cell-Specific Genes. Leukemia.

[127] Kaminskas E., Farrell A., Abraham S., Baird A., Hsieh L.S., Lee S.L., Leighton J.K., Patel H., Rahman A., Sridhara R. (2005). Approval Summary: Azacitidine for Treatment of Myelodysplastic Syndrome Subtypes. Clin. Cancer Res..

[128] Döhner H., Wei A.H., Appelbaum F.R., Craddock C., DiNardo C.D., Dombret H., Ebert B.L., Fenaux P., Godley L.A., Hasserjian R.P. (2022). Diagnosis and Management of AML in Adults: 2022 Recommendations from an International Expert Panel on Behalf of the ELN. Blood.

[129] D’Alò F., Leone G., Hohaus S., Teofili L., Bozzoli V., Tisi M.C., Rufini V., Calcagni M.L., Voso M.T. (2011). Response to 5-Azacytidine in a Patient with Relapsed Hodgkin Lymphoma and a Therapy-Related Myelodysplastic Syndrome. Br. J. Haematol..

[130] Chiappinelli K.B., Strissel P.L., Desrichard A., Li H., Henke C., Akman B., Hein A., Rote N.S., Cope L.M., Snyder A. (2015). Inhibiting DNA Methylation Causes an Interferon Response in Cancer via DsRNA Including Endogenous Retroviruses. Cell.

[131] Falchi L., Sawas A., Deng C., Amengual J.E., Colbourn D.S., Lichtenstein E.A., Khan K.A., Schwartz L.H., O’Connor O.A. (2016). High Rate of Complete Responses to Immune Checkpoint Inhibitors in Patients with Relapsed or Refractory Hodgkin Lymphoma Previously Exposed to Epigenetic Therapy. J. Hematol. Oncol..

[132] Liu Y., Wang C., Li X., Dong L., Yang Q., Chen M., Shi F., Brock M., Liu M., Mei Q. (2021). Improved Clinical Outcome in a Randomized Phase II Study of Anti-PD-1 Camrelizumab plus Decitabine in Relapsed/Refractory Hodgkin Lymphoma. J. Immunother. Cancer.

[133] Mei M.G., Chen L., Puverel S., Budde L.E., Kambhampati S., Daniels S., Dunning B., Banez M., Kwak L.W., Herrera A.F. (2023). The Combination of Nivolumab and CC-486 Is Active in Hodgkin Lymphoma Refractory to PD-1 Blockade. Blood.

[134] Huang R., Zhang X., Sophia S., Min Z., Liu X. (2018). Clinicopathological Features and Prediction Values of HDAC1, HDAC2, HDAC3, and HDAC11 in Classical Hodgkin Lymphoma. Anticancer Drugs.

[135] Younes A., Sureda A., Ben-Yehuda D., Zinzani P.L., Ong T.C., Prince H.M., Harrison S.J., Kirschbaum M., Johnston P., Gallagher J. (2012). Panobinostat in Patients with Relapsed/Refractory Hodgkin’s Lymphoma after Autologous Stem-Cell Transplantation: Results of a Phase II Study. J. Clin. Oncol..

[136] Kirschbaum M.H., Goldman B.H., Zain J.M., Cook J.R., Rimsza L.M., Forman S.J., Fisher R.I. (2012). A Phase 2 Study of Vorinostat for Treatment of Relapsed or Refractory Hodgkin Lymphoma: SWOG S0517. Leuk. Lymphoma.

[137] Janku F., Park H., Call S.G., Madwani K., Oki Y., Subbiah V., Hong D.S., Naing A., Velez-Bravo V.M., Barnes T.G. (2020). Safety and Efficacy of Vorinostat Plus Sirolimus or Everolimus in Patients with Relapsed Refractory Hodgkin Lymphoma. Clin. Cancer Res..

[138] Chase A., Cross N.C.P. (2011). Aberrations of EZH2 in Cancer. Clin. Cancer Res..

[139] Song Y., Liu Y., Li Z.M., Li L., Su H., Jin Z., Zuo X., Wu J., Zhou H., Li K. (2022). SHR2554, an EZH2 Inhibitor, in Relapsed or Refractory Mature Lymphoid Neoplasms: A First-in-Human, Dose-Escalation, Dose-Expansion, and Clinical Expansion Phase 1 Trial. Lancet Haematol..

[140] Hu X., Li J., Fu M., Zhao X., Wang W. (2021). The JAK/STAT Signaling Pathway: From Bench to Clinic. Signal Transduct. Target. Ther..

[141] Tiacci E., Ladewig E., Schiavoni G., Penson A., Fortini E., Pettirossi V., Wang Y., Rosseto A., Venanzi A., Vlasevska S. (2018). Pervasive Mutations of JAK-STAT Pathway Genes in Classical Hodgkin Lymphoma. Blood.

[142] Weniger M.A., Melzner I., Menz C.K., Wegener S., Bucur A.J., Dorsch K., Mattfeldt T., Barth T.F.E., Möller P. (2006). Mutations of the Tumor Suppressor Gene SOCS-1 in Classical Hodgkin Lymphoma Are Frequent and Associated with Nuclear Phospho-STAT5 Accumulation. Oncogene.

[143] Fernández S., Solórzano J.L., Díaz E., Menéndez V., Maestre L., Palacios S., López M., Colmenero A., Estévez M., Montalbán C. (2023). JAK/STAT Blockade Reverses the Malignant Phenotype of Hodgkin and Reed-Sternberg Cells. Blood Adv..

[144] Kim S.J., Yoon D.H., Kang H.J., Hong J.Y., Lee H.S., Oh S.Y., Shin H.J., Kong J.H., Yi J.H., Sakamoto K. (2019). Ruxolitinib Shows Activity against Hodgkin Lymphoma but Not Primary Mediastinal Large B-Cell Lymphoma. BMC Cancer.

[145] Van Den Neste E., André M., Gastinne T., Stamatoullas A., Haioun C., Belhabri A., Reman O., Casasnovas O., Ghesquieres H., Verhoef G. (2018). A Phase II Study of the Oral JAK1/JAK2 Inhibitor Ruxolitinib in Advanced Relapsed/Refractory Hodgkin Lymphoma. Haematologica.

[146] Gillessen S., Pluetschow A., Vucinic V., Ostermann H., Kobe C., Bröckelmann P.J., Böll B., Eichenauer D.A., Heger J.M., Borchmann S. (2022). JAK Inhibition with Ruxolitinib in Relapsed or Refractory Classical Hodgkin Lymphoma: Final Results of a Phase II, Open Label, Multicentre Clinical Trial (JeRiCHO). Eur. J. Haematol..

[147] Bachanova V., Hegerova L., Cao Q., Janakiram M., Maakaron J., Ayyappan S., Weisdorf D.J., Zak J., Farooq U., Kenkre V.P. (2021). Ruxolitinib Plus Nivolumab in Patients with R/R Hodgkin Lymphoma after Failure of Check-Point Inhibitors: Preliminary Report on Safety and Efficacy. Blood.

[148] Svoboda J., Barta S.K., Landsburg D.J., Dwivedy Nasta S., Hwang W.-T., Delp G., Amundsen B., Ballard H.J., Gerson J.N., Chong E.A. (2020). Everolimus Plus Itacitinib in Relapsed/Refractory Classical Hodgkin Lymphoma: Results of a Phase I/II Investigator Initiated Trial (EVITA Study). Blood.

[149] Cappell K.M., Kochenderfer J.N. (2023). Long-Term Outcomes Following CAR T Cell Therapy: What We Know so Far. Nat. Rev. Clin. Oncol..

[150] Benevolo Savelli C., Clerico M., Botto B., Secreto C., Cavallo F., Dellacasa C., Busca A., Bruno B., Freilone R., Cerrano M. (2023). Chimeric Antigen Receptor-T Cell Therapy for Lymphoma: New Settings and Future Directions. Cancers.

[151] Wang C.M., Wu Z.Q., Wang Y., Guo Y.L., Dai H.R., Wang X.H., Li X., Zhang Y.J., Zhang W.Y., Chen M.X. (2017). Autologous T Cells Expressing CD30 Chimeric Antigen Receptors for Relapsed or Refractory Hodgkin Lymphoma: An Open-Label Phase I Trial. Clin. Cancer Res..

[152] Ramos C.A., Grover N.S., Beaven A.W., Lulla P.D., Wu M.F., Ivanova A., Wang T., Shea T.C., Rooney C.M., Dittus C. (2020). Anti-CD30 CAR-T Cell Therapy in Relapsed and Refractory Hodgkin Lymphoma. J. Clin. Oncol..

[153] Ahmed S., Flinn I.W., Mei M., Riedell P.A., Armand P., Grover N.S., Balyan R., Ding C., Myo A., Horak I.D. (2022). Updated Results and Correlative Analysis: Autologous CD30.CAR-T-Cell Therapy in Patients with Relapsed or Refractory Classical Hodgkin Lymphoma (CHARIOT Trial). Blood.

[154] Voorhees T.J., Zhao B., Oldan J., Hucks G., Khandani A., Dittus C., Smith J., Morrison J.K., Cheng C.J., Ivanova A. (2022). Pretherapy Metabolic Tumor Volume Is Associated with Response to CD30 CAR T Cells in Hodgkin Lymphoma. Blood Adv..

[155] Voorhees T.J., Beaven A.W., Dittus C., Hucks G.E., Morrison J.K., Cheng C.J.A., Cavallo T., Park S.I., Dotti G., Serody J.S. (2022). Clinical Activity of Anti-PD-1 Therapy Following CD30 CAR-T Cell Therapy in Relapsed Hodgkin Lymphoma. Blood.

[156] Dave H., Terpilowski M., Mai M., Toner K., Grant M., Stanojevic M., Lazarski C., Shibli A., Bien S.A., Maglo P. (2022). Tumor-Associated Antigen-Specific T Cells with Nivolumab Are Safe and Persist in Vivo in Relapsed/Refractory Hodgkin Lymphoma. Blood Adv..

[157] Jones R.J., Gocke C.D., Kasamon Y.L., Miller C.B., Perkins B., Barber J.P., Vala M.S., Gerber J.M., Gellert L.L., Siedner M. (2009). Circulating Clonotypic B Cells in Classic Hodgkin Lymphoma. Blood.

[158] Beatty G.L., Haas A.R., Maus M.V., Torigian D.A., Soulen M.C., Plesa G., Chew A., Zhao Y., Levine B.L., Albelda S.M. (2014). Mesothelin-Specific Chimeric Antigen Receptor MRNA-Engineered T Cells Induce Anti-Tumor Activity in Solid Malignancies. Cancer Immunol. Res..

[159] Svoboda J., Rheingold S.R., Gill S.I., Grupp S.A., Lacey S.F., Kulikovskaya I., Suhoski M.M., Joseph Melenhorst J., Loudon B., Mato A.R. (2018). Nonviral RNA Chimeric Antigen Receptor-Modified T Cells in Patients with Hodgkin Lymphoma. Blood.

